# Bleomycin induces fibrotic transformation of bone marrow stromal cells to treat height loss of intervertebral disc through the TGFβR1/Smad2/3 pathway

**DOI:** 10.1186/s13287-020-02093-9

**Published:** 2021-01-07

**Authors:** Xiao Yang, Zhiqian Chen, Chen Chen, Chen Han, Yifan Zhou, Xunlin Li, Haijun Tian, Xiaofei Cheng, Kai Zhang, An Qin, Tangjun Zhou, Jie Zhao

**Affiliations:** Shanghai Key Laboratory of Orthopedic Implants, Department of Orthopedics, Ninth People’s Hospital, Shanghai Jiaotong University School of Medicine, 639 Zhizaoju Road, Shanghai, 200011 People’s Republic of China

**Keywords:** Intervertebral disc degeneration, Bleomycin, Bone marrow stromal cells, Annulus Fibrosus cells, Reparative fibrosis, TGFβ-TGFβR1-SMAD2/3 signaling pathway

## Abstract

**Background:**

Lower back pain is often accredited to loss of intervertebral disc (IVD) height and compromised spine stability as a result of intervertebral disc degeneration (IVDD). We aim to locally use bleomycin to induce the fibrotic transformation of bone marrow stromal cells (BMSCs) as a means to induce reparative fibrosis to slow down the height loss.

**Methods:**

IVDs from patients were gathered for histological examination. The expression of the transforming growth factor beta 1 (TGF-β) signaling pathway was determined by qPCR and western blotting. Nucleus pulposus (NP) cells, annulus fibrosus (AF) cells, and the rats’ bone marrow stromal cells (BMSC) were cultured and their responsiveness to bleomycin was evaluated by Cell Counting Kit-8, comet assay, transwell migration, and wound healing assays. Rat IVDD models were created by puncture and rescued by bleomycin injection, and the effectiveness was evaluated by images (X-ray and MRI) and atomic force microscope.

**Results:**

Histological examination showed increased levels of pro-fibrotic markers in IVDD tissues from patients. AF cells and BMSC cells were induced to adopt a pro-fibrotic phenotype with increased expression fibrotic markers Col1a1, Col3a1, and FSP1. The pro-fibrotic effect of bleomycin on AF cells and BMSCs was in part due to the activation of the TGFβ-TGFβR1-SMAD2/3 signaling pathway. Pharmacological inhibition or gene knock-down of TGFβR1 could mitigate the pro-fibrotic effects.

**Conclusion:**

Locally, injection of bleomycin in rats’ IVD induced rapid fibrosis and maintained its height through the TGFβ-TGFβR1-SMAD2/3 signaling pathway.

## Background

Low back pain and other spine disorders due to intervertebral disc degeneration affect over 600 million people worldwide, imposing a substantial burden on the medical and socioeconomic structures of all developed countries [1]. Intervertebral disc degeneration is a complicated process involving three main tissues: the nucleus pulposus (NP), a proteoglycan-rich gelatinous center of the intervertebral disc; the outer and inner annulus fibrosus (AF), a partially concentric collagen-rich fibrocartilaginous tissue surrounding the NP; and two cartilaginous endplates that interface with the vertebral bodies superiorly and inferiorly [2, 3]. Intervertebral disc degeneration is a progressive, cell-mediated cascade of molecular, structural, and biomechanical changes: degeneration starts in the NP where the reduction in proteoglycan content and a decrease in the ratio of proteoglycan to collagen result in the loss of hydrostatic properties leading to structural wear and compromised biomechanical functions [4]. Continued dehydration results in the collapse of the NP, loss of intervertebral disc height, and the gradual loss of NP-AF borders ultimately progressing to complete degeneration of the entire intervertebral disc. This causes spine instability and nerve root compression evoking chronic back pain [4, 5].

During degeneration in discs, ruptured NP and AF would recruit multiple cells to finish the repair process, of which the most important cells are fibroblastic cells and MSCs [6], but due to the low cell population and lack of nutrient supply to the intervertebral disc tissues, self-regeneration is limited [5, 7, 8]. Conventional treatment strategies are largely limited to symptomatic relief with limited long-term efficacy [9–11], and this spinal fusion surgery does not restore biomechanical properties but may in fact induce degeneration of adjacent vertebral bodies due to the increased mechanical stress they have to sustain [12]. A cell-based approach has also been studied in the past few years, and one of the most focused methods is disc regeneration via MSC transplantation [13–15]. Moreover, recent researches have unveiled that there are some MSCs distributed around the outer AF region and endplate [16]. So, from a different perspective that a former approach of our studies by injection of autologous dermal fibroblast cells into degenerative intervertebral to treat IVDD [17], we showed that the induction of reparative fibrosis combined with two essential cells in discs, one is to induce the fibrotic phenotype of MSCs and the other is to promote the fibroblastic-like cell AF cells.

Bleomycin is a cytotoxic chemotherapeutic compound used in the treatment of lymphoma, leukemia, squamous cell carcinomas, and some genital tract tumors. Its major side effect as an anti-cancer agent is the induction of fibrosis particularly pulmonary fibrosis. Interestingly, despite the mechanism of DNA damage to treat cancer, Lin et al. used its pro-fibrotic function to induce the fibrosis of extracranial arteriovenous malformation by intralesional interstitial bleomycin injection, just like a sclerosant [18]. Their prospective studies involved 34 patients and, finally, successfully cure 27 of them making an extraordinary result, and this local injection therapy was once a month with a low dose less than 400 mg or 5 mg/kg, which contributed to less side effects [19, 20]. Based on these studies, we hypothesized that the pro-fibrotic effects of bleomycin injected locally could on the one hand induce the fibrosis of MSCs and AF cells to maintain the disc height; on the other hand, it could accelerate the absorption of herniated NP in degenerative intervertebral discs.

In this study, we investigated the potential use of bleomycin to induce reparative fibrosis as a means to maintain intervertebral disc height, and we tried to use it to induce the fibrosis phenotype of BMSCs and offer an alternative treatment in degeneration caused height loss. We also explored the potential molecular mechanism underlying the effects of bleomycin particularly in terms of the TGFβ-SMAD2/3 signaling, one of the key pathways in the fibrotic process.

## Methods

### Intervertebral discs specimens

**Fig. 1:**
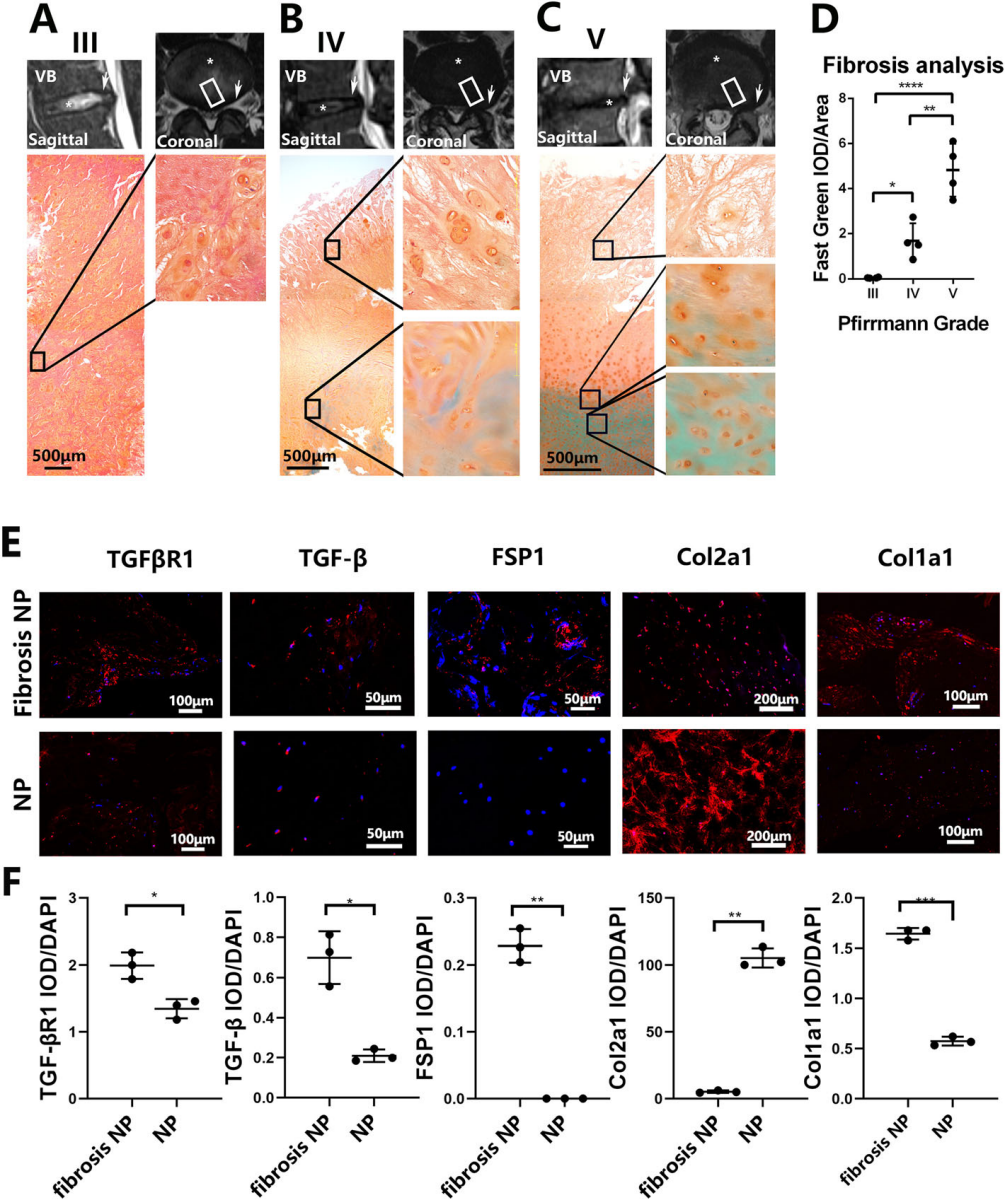
Human intervertebral disc undergone fibrosis with degeneration. **a**–**c** Intervertebral disc samples were gathered, and their paraffin section was made to undergo Safranin O-Fast Green stain. Asterisks indicate the nucleus pulposus region of the disc, arrows indicate the approach to dissect the discs, boxes indicate the area of samples to be stained. **d** IOD levels of the green region were analyzed by IPP.6.0 and subsequently calculated with Graphpad8.0 by ordinary one-way ANOVA test. **e** Immunofluorescence assay of TGFβ, TGFβR1, FSP1, Col2a1, and Col1a1 in the NP region and fibrosis NP region described in **a**– **c**. **f** IOD levels of the red region were analyzed by IPP.6.0 and subsequently calculated with Graphpad8.0 by Student *t* test. All data are presented as mean ± sd. from three experiments. *P < 0.05, **P < 0.01, ***P < 0.001, and ****P < 0.0001

Intervertebral discs (≥ 5 mm) were obtained from 12 patients, including 7 males and 5 females with a combined average age of 52.8 ± 12.8 years (age range of 30–74 years) and diagnosed with lumbar disc herniation (LDH) or degenerative lumbar spondylolisthesis (DLS) (Fig. 1a–c; Table 1). The NP were sampled from L4/5 and subjected to histological and immunohistochemical analyses. Intervertebral disc degeneration was evaluated using the Pfirrmann grading system.

**Table 1:** Patient information

Name	Pfirrmann grade	Gender	Age	Diagnosis
Z	3	Male	49	LDH
G	4	Male	51	LDH
S	5	Female	62	DLS
T	3	Male	56	LDH
T	5	Male	62	LDH
Zh	4	Female	53	LDH
L	4	Female	65	DLS
W	3	Male	30	LDH
L	5	Male	74	LDH
Y	5	Female	39	LDH
M	4	Male	57	LDH
Ya	3	Female	36	LDH

### Isolation and cell culture of BMSCs

Six-week-old male Sprague-Dawley rats (Shanghai Lab, Animal Research Center Co. Ltd., Shanghai, China) were killed by cervical dislocation and soaked in 75% ethanol for 10 min. Extraction and isolation of BMSCs from the two lower legs of SD rats were done with sterile operation, BMSCs were cultured in Minimum Essential Medium α (MEMα) supplemented with 10% FBS and 1% penicillin-streptomycin (Gibco, Thermo Fisher Scientific, Waltham, MA, USA).

### Culture of NP and AF cell lines

The rat’s NP and AF cells are immortalized cell lines [21] which were kindly gifted by Dr. Chen Di at the Department of Orthopedic Surgery, Rush University Medical Center (Chicago, IL, USA). Cells were maintained in Dulbecco’s modified Eagle’s medium (DMEM) supplemented with 10% FBS and 1% penicillin-streptomycin (Gibco, Thermo Fisher Scientific, Waltham, MA, USA).

### RNA extraction and real-time quantitative PCR analyses

Total RNA was isolated from tissues and cells using TRIzol Reagent (Thermo Fisher Scientific, Waltham, MA, USA) as per the manufacturer’s protocol. First-strand complementary DNAs (cDNAs) were reversed transcribed from extracted RNAs using the cDNA Synthesis Kit (Takara Bio, Otsu, Japan). Relative mRNA expression was determined by RT-PCR using the GoTaq 1-step RT-qPCR System (Promega, Madison, WI, USA) followed by agarose gel electrophoresis (Bio-Rad Laboratories, Hercules, CA, USA). Real-time qPCR was conducted using the TB Green Premix Ex Taq Kit (Takara Bio) on an Applied Biosystems QuantStudio 6 Flex Real-Time PCR System (Thermo Fisher Scientific). Specific primer pairs were designed using NCBI BLAST and sequences provided in Table 2. The gene expression of GAPDH or β-actin was used as an internal control. Target gene expression levels were determined using the 2^−ΔΔCT^ method. The mean CT value of target genes in the experimental groups was normalized to the CT value of GAPDH or β-actin to give a ΔCT value. This was then further normalized to control samples to obtain ΔΔCT.

**Table 2:** PCR primers information

Gene	Accession number	Description	5′-primer-3′
Fn1	NM_019143.2	F R	GGATCCCCTCCCAGAGAAGT GGGTGTGGAAGGGTAACCAG
Col1a1	NM_053304.1	F R	GGATCGACCCTAACCAAGGC GATCGGAACCTTCGCTTCCA
Col3a1	NM_032085.1	F R	AGTGGCCATAATGGGGAACG CACCTTTGTCACCTCGTGGA
TGFβR1	NM_012775.2	F R	TGCCTGCTTCTCATCGTGTT TGCTTTTCTGTAGTTGGGAGT
TGFβR2	NM_031132.3	F R	CCAAGTCGGTTAACAGCGAT TGAAGCCGTGGTAGGTGAAC
TGFβR3	NM_017256.1	F R	GAGGGGCTGTCACTTACCAC CTAATCCCCTCGCTGACCAC
TGFβ1	NM_021578.2	F R	CACTCCCGTGGCTTCTAGTG GGACTGGCGAGCCTTAGTTT
MMP3	NM_133523.3	F R	TTTGGCCGTCTCTTCCATCC GCATCGATCTTCTGGACGGT
MMP13	NM_133530.1	F R	TGCTGCATACGAGCATCCAT TGTCCTCAAAGTGAACCGCA
Timp1	NM_012886.2	F R	AAATACCTTCCTGGTCCCGC TAGGAGACTCTGGCGTCTGT
P21	NM_017198.1	F R	TCCTCTCGGCTATTACCGGC GGTGTTTCTCATCGGAGGGG
CDK1	NM_019296.1	F R	GGAACAGAGAGGGTCCGTTG GCACTCCTTCTTCCTCGCTT
CDK2	NM_199501.1	F R	TGCTTATCAACGCAGAGGGG GGGTCACCATTTCGGCAAAG
CDK4	NM_053593.2	F R	GGACCGATCCCCGGTGTA TCAGGTCCCGGTGAACAATG
CDK6	NM_001191861.1	F R	TCGTGGAAGTTCAGACGTGG CGACAGGTGAGAATGCAGGT
CCNG2	NM_001105725.2	F R	ACCTGTGTGAAAGCGGAACA TGGCACCCATGTACAACACA
CCND2	NM_022267.1	F R	GATGATCGCAACTGGAAGCG TGGTCCGGATCTTCCACAGA

### Cell viability analysis

Cell viability following bleomycin treatment was evaluated using the Cell Counting Kit-8 (CCK-8; Dojindo Laboratories Co., Ltd., Kumamoto, Japan). Cells seeded onto 96-well plates at a density of 8 × 10^3^ cells/well the day before were treated with increasing concentrations of bleomycin sulfate (1, 5, and 10 μg/ml, dissolved in PBS; Selleck Chemicals, Houston, TX, USA) for 24, 48, 72, and 96 h. NP, AF, and BMSC cells were cultured in DMEM, DMEM/F12, or MEMα, respectively, all supplemented with 10% FBS and 1% penicillin/streptomycin (complete DMEM or complete DMEM/F12). Cell media containing bleomycin were changed every 2 days. At the end of the experimental periods, cells were incubated with fresh complete media containing 10 μl of CCK-8 reagent for 1 h at 37 °C. Complete media containing CCK-8 reagent but no cells and untreated cells were used as blank and mock controls, respectively. The absorbances (measured as optical density (OD)) at 450 nm were measured on an Infinite M200 Pro multimode microplate reader (Tecan Life Sciences, Männedorf, Switzerland). ODs of the bleomycin-treated groups were normalized to corresponding blank ODs to account for background interference.

### TGFβR1 siRNA knockdown

AF cells seeded onto 6-well plates at a density of 1 × 10^5^ cells/well the day before were transfected with small interfering RNA (siRNA) against TGFβR1 (siTGFβR1: sense 5′-GGAGAUUGUUGGUACCCAAGG-3′ and anti-sense 5′-UUGGGUACCAACAAUCUCCAU-3′) or with a scrambled siRNA control (NC: sense 5′-UUCUCCGAACGUGUCACGUTT-3′ and anti-sense 5′-ACGUGACACGUUCGGAGAATT-3′) (IBSBIO, Shanghai, China) using Lipofectamine 3000 transfection reagent in accordance with the manufacturer’s protocol. After 6 h, the media containing transfection reagent and siRNAs were removed and replaced with fresh complete media. Twenty-four hours post-transfection, cells were harvested for total protein or total RNA extraction.

### Comet analysis (single-cell gel electrophoresis)

The method for comet analysis or single-cell gel electrophoresis (SCGE) was performed as previously described [22, 23] Briefly, cultured AF cells, NP cells, and BMSCs were trypsinized, centrifuged, and resuspended in 1 × PBS (Ca^2+^ and Mg^2+^ free; Gibco, Thermo Fisher Scientific) to a final cell density of 1 × 10^5^ cells/ml. Next, 50 μl of cell suspensions was then mixed with 500 μl low melting point (LMP) agarose (1% at 37 °C, Comet SCGE Assay Kit; Enzo Life Sciences, Farmingdale, NY, USA), and then 75 μl of the cell-agarose mixture was spread onto glass slides precoated with 1% normal melting point (NMP) agarose and allowed to settle for 10 mins at 4 °C in the dark. Slides were then immersed in pre-chilled Lysis Solution (Comet SCGE Assay Kit; Enzo Life Sciences) and incubated on ice for 1 h. After lysis, the slides were placed on a horizontal gel electrophoresis unit containing electrophoretic Alkaline Solution (Comet SCGE Assay Kit; Enzo Life Sciences) for 60 min at room temperature in the dark to allow the DNA to unwind. Electrophoresis was then carried out for 10 min at 25 V and 300 mA (0.73 V/cm). After electrophoresis, the slides were rinsed in distilled water, placed in neutralization solution (pH 7.5) to remove the alkali and detergent, then dehydrated with 70% ethanol for 5 min and air-dried. Immediately before examination, the slides were stained with 100 μl of ethidium bromide (10 μg/ml) for 30 min at room temperature in the dark. Comets were visualized under × 400 magnification using epifluorescence microscopy (Leica DM4000 B; Leica Microsystems, Wetzlar, Germany).

### Scratch-wound healing assay

The scratch-wound healing assay was performed to examine the effects of bleomycin on collective migration and recolonization in a 2-dimensional environment. AF cells with or without TGFβR1 siRNA knockdown and BMSCs were cultured to 100% confluence. Under sterile conditions, a linear scratch line was made straight down the center of the cell monolayer using the tip of a sterilized 200-μl micropipette tip. Cell media were carefully aspirated to remove cellular debris and floating cells and then replaced with fresh serum-free DMEM/F12 or MEMα without or with bleomycin (5 or 10 μg/ml) ± TGFβR1 inhibitor (LY364947, 10 μM; Selleck, USA). Phase-contrast images were captured of the initial scratch wound for reference and designated time 0. Further images were captured at 24 and 48 h, and the distance between the leading cell edges at each time point was measured using the ImageJ software (National Institutes of Health, USA).

### Millicell transwell migration assay

The effects of bleomycin on single-cell migration through a three-dimensional environment were examined using the transwell migration assay (Millicell Standing Cell Culture Inserts, 8-μm pore size; Merck-Millipore, Burlington, MA, USA). Briefly, AF cells with or without TGFβR1 siRNA knockdown and BMSCs were seeded into the upper chamber of the Millicell Standing Cell Culture inserts at a density of 8 × 10^4^ cells/well in serum-free DMEM/F12 or MEMα media without or with bleomycin (5 or 10 μg/ml) ± TGFβR1 inhibitor (LY364947, 10 μM; Selleck, USA). The inserts were then placed into 24-well plates filled with complete media (FBS as chemoattractant source) and cultured for 24 h. At the end of the experiment, the media in the inserts were discarded, and adherent cells were fixed in 4% paraformaldehyde (PFA) for 30 min and then stained with 0.2% crystal violet for 5 min. Cells adhering to the membrane inside the inserts (i.e., cells that have not migrated) were gently removed using a cotton-tipped applicator. Migrated cells on the other side of the inserts were imaged under a light microscope (Leica DM4000 B; Leica Microsystems) and staining intensity analyzed with the Image-Pro Plus 6.0 software to evaluate the ratio of integrated optical density (expressed as the IOD/area for each sample).

### Assessment of apoptosis and cell cycles by flow cytometry

The effects of bleomycin were evaluated using flow cytometry following staining with APC-Annexin V and propidium iodide (PI) for cell apoptosis or just PI for cell cycles based on apoptosis staining kit (Thermo Fisher Scientific) and PI/RNase Staining Buffer (BD Pharmingen) according to the manufacturer’s protocol. Cell suspensions were subjected to flow cytometry on a FACSCalibur Flow Cytometer (BD Biosciences) counting at least 10,000 events. The apoptotic rate was quantified based on the percentage of cells in the right upper (Q2; positive staining for APC-Annexin V and PI) and right lower (Q3; positive staining for APC-Annexin V and negative for PI) quadrant of the flow cytometric scatterplot.

### Senescence assays

NP cells and BMSCs were identified using the Senescence β-Galactosidase Staining Kit (Beyotime Biotechnology) according to the manufacturer’s protocol. NP cells and BMSCs were seeded onto a 12-well plate at a density of 3 × 10^6^ cells/well, following with 5 μg/ml bleomycin once or three times, every treatment period lasts for 3 days. Then, cells were fixed with Fixative Solution for 15 min at RT and then incubated with β-Galactosidase Staining buffer at 37 °C overnight in a dry incubator without CO_2_. Then, the cell percentage of positive cells was calculated.

### Western blot analysis

Total cellular proteins were extracted from cultured cells using RIPA lysis buffer supplemented with phosphatase and protease inhibitors (Roche, Basel, Switzerland). Equal quantities of extracted proteins (20–30 μg) were resolved on 10% or 12.5% SDS-PAGE gel and separated proteins electroblotted onto 0.22-μm PVDF membranes (Merck-Millipore). The membranes were blocked with 5% BSA-PBS at room temperature for 1 h and then incubated with primary antibodies (diluted 1:1000 in 5% BSA-PBS) overnight (at least 16 h) at 4 °C. Primary antibodies against SMAD2 (Ser308, D43B4; rabbit mAb), phospho-SMAD2 (Ser465/467, 138D4; rabbit mAb), SMAD3 (C67H9; rabbit mAb), phospho-SMAD3 (Ser423/425, C25A9; rabbit mAb), SMAD2/3 (D7G7; rabbit mAb), phosphor-SMAD2/3 (Ser465/467; Ser423/425; rabbit mAb), SMAD4 (D3M6U; rabbit mAb), PARP (#9542; rabbit mAb), cleaved PARP (D64E10; rabbit mAb), and β-actin (D6A8; rabbit mAb) were purchased from Cell Signaling Technology (Danvers, MA, USA). Primary antibodies against FSP1 (S100A4; rabbit mAb), TGFβ receptor I (ab31013; rabbit mAb), type I collagen (ab6308; rabbit mAb), TGFβ1 (ab64715; rabbit mAb), P21 (ab109199; rabbit mAb), and P53 (ab26; mouse pAb) were obtained from Abcam (Cambridge, UK). The membranes were then washed extensively in Tris-buffered saline Tween20 (TBST) and subsequently incubated with anti-rabbit IgG (H+L) (DyLight™ 800 4× PEG Conjugate; Cell Signaling Technology) secondary antibody (1:5000 dilution) for 1 h at room temperature in the dark. The membranes were again extensively washed in TBST, and protein immunoreactivity was detected on a LI-COR Odyssey Fluorescence Imaging System (LI-COR Biosciences, Lincoln, NE, USA). Semi-quantitative analysis of protein immunoreactive band intensity was measured using the Image-Pro Plus 6.0 software and normalized to the internal loading control β-actin.

### Animals and surgical procedures

All animal experimentation was approved by the Institutional Animal Care and Ethics Committee of the Ninth People’s Hospital, Shanghai Jiaotong University School of Medicine (Shanghai, China), and performed in accordance with the principles and procedures of the National Institutes of Health (NIH) Guide for the Care and Use of Laboratory Animals and the Guidelines for Animal Treatment of Shanghai Jiaotong University. Six 8-week-old male Sprague-Dawley rats (Shanghai Lab, Animal Research Center Co. Ltd., Shanghai, China) were housed under pathogen-free conditions at 26–28 °C and 50–65% humidity with 12-h day/night cycle. Animals were fed standard rodent chow and had access to freshwater ad libitum. Before surgical procedures, rats were anesthetized by intraperitoneal injections of pentobarbital sodium (5 mg/100 g of body weight). The tails were sterilized with iodinated polyvinylpyrrolidone, and then a ventral longitudinal skin incision was made over the tail to reveal the intervertebral disc at coccyx vertebrae 6–10. The intervertebral discs at Co6/7 were used as sham controls, and the intervertebral discs at Co7/8, Co8/9, and Co9/10 were used as the experimental groups. Intervertebral discs were punctured with a 20-gauge sterile needle oriented perpendicular to the skin to make ensure insertion at the center of the disc level through the AF into the NP. The incision was then sutured, and rats were allowed post-operative recovery for 2 weeks. A group of mice (*n* = 3) was sacrificed, and the tails extracted, cleaned of soft tissues, and the vertebral column fixed in 4% PFA. To the remaining rats (*n* = 3), surgical exposure of the intervertebral discs at Co8/9 and Co9/10 was again carried out, and 5 μl of bleomycin at concentrations of 10 and 5 μg/ml was injected respectively into each disc. The incision was sutured, and rats were allowed 2 and 4 weeks of post-treatment recovery. At the end of the experimental period, all remaining rats were sacrificed, and the tails extracted, cleaned of soft tissues, and the vertebral column fixed in 4% PFA.

### Histology and immunofluorescence staining

Fixed intervertebral disc tissue samples were embedded into the paraffin blocks then subjected to histological sectioning (5 μm thickness). For histological assessment, the paraffin tissue sections were processed for Safranin O-Fast Green and Sirius Red staining in accordance with the standard laboratory protocols. For immunofluorescence assessment, BMSCs were cultured in a slide with a confluence of 10% and fixed with 4% PFA, then these cell slides are with tissue sections to be de-paraffinized in graded xylene, rehydrated in graded alcohol solutions, and then incubated in antigen retrieval buffer (Roche) at 37 °C for 30 min. After cooling to room temperature, the slides were immersed in PBS (pH 7.4) and washed 3 times for 5 min each. Auto-fluorescence quencher was added to the sections for 5 min and then blocked with blocking buffer for 30 min at room temperature. The sections were subsequently incubated with primary antibodies in a wet box at 4 °C overnight. Primary antibodies were used at 1:100 dilution and included anti-Col1a1, anti-Col2a1, anti-FSP1, anti-TGFβ, anti-TGFβR1, S100A9 (all purchased from Cell Signaling Technology), and anti-Keratin 18(abs130128, absin). The next day, the sections were washed with PBS and then incubated with Alexa Fluor 594 Conjugate secondary antibody (anti-rabbit, 1:500; Cell Signaling Technology) for 50 min at room temperature in the dark. The sections were washed with PBS and then incubated with DAPI solution (Sigma-Aldrich, St. Louis, MO, USA) for 10 min in the dark to stain the cell nuclei. The sections were subjected to the final PBS washes, air-dried, and then sealed with anti-fluorescence-quenching tablets. Digital fluorescence images were captured under a Leica DM4000 B epifluorescence microscope (Leica Microsystems) and IOD measurements carried out using the Image-Pro Plus 6.0 software.

### Radiographic and magnetic resonance imaging analysis

Digital X-ray imaging of the punctured intervertebral discs was conducted in the anteroposterior axis with a 21-lp/mm detector that provides up to × 5 geometric magnification (Faxitron VersaVision; Faxitron Bioptics LLC, Tucson, AZ, USA). MRI imaging of the same punctured intervertebral discs was carried out on a Siemens Magnetom Prisma E11 (Siemens Healthineers, Erlangen, Germany) with the following parameters: TR 3000 ms, TE 80 ms, 1.1 mm thickness, 0.22 mm interval, FOV 160 × 65 mm, and voxel size 0.25 × 0.25 × 1.1 mm.

### Atomic force microscopy

For atomic force microscopy (AFM), the extracted punctured vertebrae were dissected to make a paraffin section, and nanoindentation was performed on a Park NX20 (Park Systems, South Korea) equipped with microspherical colloidal tips (*R* < 10 nm, nominal *k* ≈ 0.2 N/m, Tip:Si/Tipless/Top, cantilever Si/AI/Top; Park Systems). For a wide range of undulating surfaces, the scanning rate of 13 Hz was used. A large scanning rate can reduce drift, but it is generally only used for scanning small flat surfaces. Indentation was applied at a z-piezo displacement rate of 10 μm/s to a maximum load of ~ 120 nN using a Scan Asyst-Air probe, a curvature radius of 5 nm, and a force constant of 0.4 N/m. Young’s modulus, adhesion force, and deformation parameters were evaluated.

### Statistical analysis

Three independent experiments or repeated measurements were conducted for all data. Data are presented as the mean ± standard deviation (S.D.). Significance differences between the study groups were obtained by Student’s *t* test or one-way analysis of variance (ANOVA) using the SPSS 19.0 software (IBM Corporation, Armonk, NY, USA). Statistical significance was set if the *p* value < 0.05 unless otherwise indicated.

## Results

### Intervertebral disc degeneration correlates with fibrotic changes in NP in patients with lumbar disc herniation or degenerative lumbar spondylolisthesis

Degenerative and fibrotic changes in the intervertebral discs from 12 patients (patient details presented in Table 1) with lumbar disc herniation (LDH) or degenerative lumbar spondylolisthesis (DLS) were examined by MRI and Safranin O-Fast Green (SOFG) histological staining (Fig. 1a–c). Pfirrmann grading based on MRI scans [5, 9] was then correlated with the degree of fibrosis based on IOD of SOFG staining in the NP tissues of the intervertebral disc (Fig. 1d). Consistent with the Pfirrmann grading for intervertebral disc degeneration, the higher the Pfirrmann grade, the greater the degree of the NP fibrosis in the intervertebral discs as demonstrated by increasing IOD score of Fast Green staining in the tissue sections (Fig. 1d). That is, a higher Pfirrmann grade is correlated with greater fibrotic changes in the NP tissues. We further noticed that with higher Pfirrmann grade such as IV and V (severe intervertebral disc degeneration with or without collapsed disc space), the fibrotic changes occurs closer to the center of the NP tissues in the intervertebral disc (Fig. 1b–d). No fibrosis was seen in intervertebral discs that were presented as Pfirrmann grade III (Fig. 1a, d). We also examined the expression of fibrotic protein markers between the non-fibrotic and fibrotic regions of the NP tissues (Fig. 1e, f). Compared with the non-fibrotic region, the fibrotic region expressed high levels of protein markers involved in fibrosis such as Col1a1, FSP1, TGFβ, and TGFβR1, and markedly decreased levels of Col2a1 (Fig. 1e, f). Of these markers, FSP1 or fibroblast-specific protein-1 is highly expressed in fibroblasts and often used as a fibrotic biomarker. The elevated expression of TGFβ and TGFβR1 suggests the involvement of the TGFβ signaling pathway in inducing fibrotic changes in the NP region during intervertebral disc degeneration.

### BMSCs were induced to acquire fibrotic phenotype by bleomycin in vitro

**Fig. 2:**
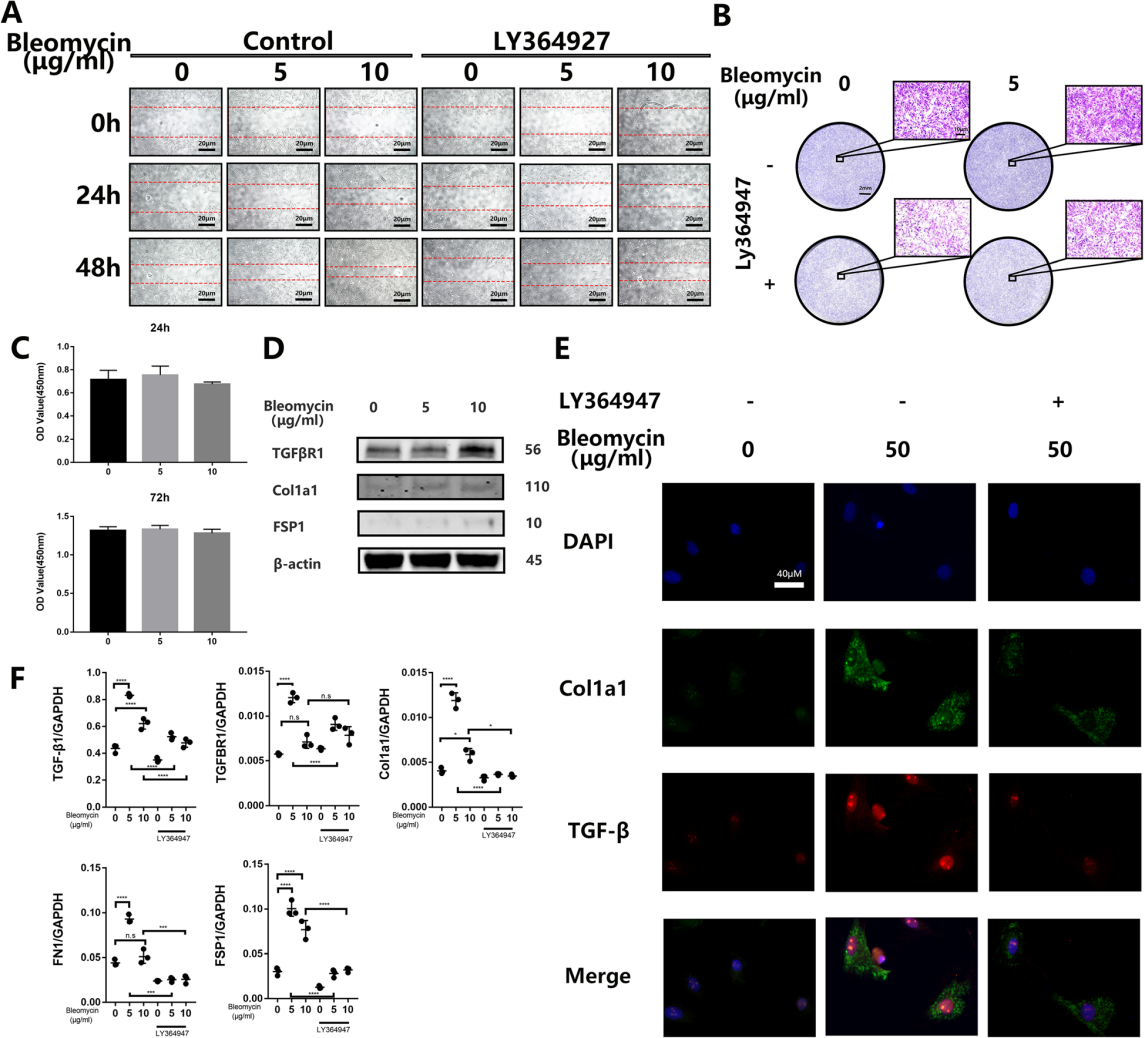
BMSCs were induced to acquire fibrotic phenotype by bleomycin in vitro. **a**, **b** BMSCs were pretreated with TGFβR1 inhibitor LY364947(10 μM) or DMSO and stimulated with bleomycin in a concentration of 0, 5, and 10 μg/ml, then cells were cultured for 48 h and observed in a checkpoint of 0, 24 h, and 48 h to calculate the migration rate by wound healing assay or cultured for 24 h to do the transwell test. **c** BMSCs were stimulated with bleomycin in different concentrations of 0, 5, and 10 μg/ml in 24 and 48 h, following cell viability analysis using Cell Count Kit-8 and then subsequently calculated with Graphpad8.0 by ordinary one-way ANOVA test. **d** Western blot analysis of TGFβR1, FSP1, and Col1a1 in BMSCs uses β-actin as a reference. **e** Immunofluorescence assay of TGFβ and Col1a1 in the BMSCs. **f** Q-PCR analysis of the relative mRNA expression levels of TGFβ, TGFβR1, Fn1, FSP1, and Col1a1 in BMSCs with bleomycin. All data are presented as mean ± sd. from three experiments. *P < 0.05, **P < 0.01, ***P < 0.001, and ****P < 0.0001

**Fig. 3:**
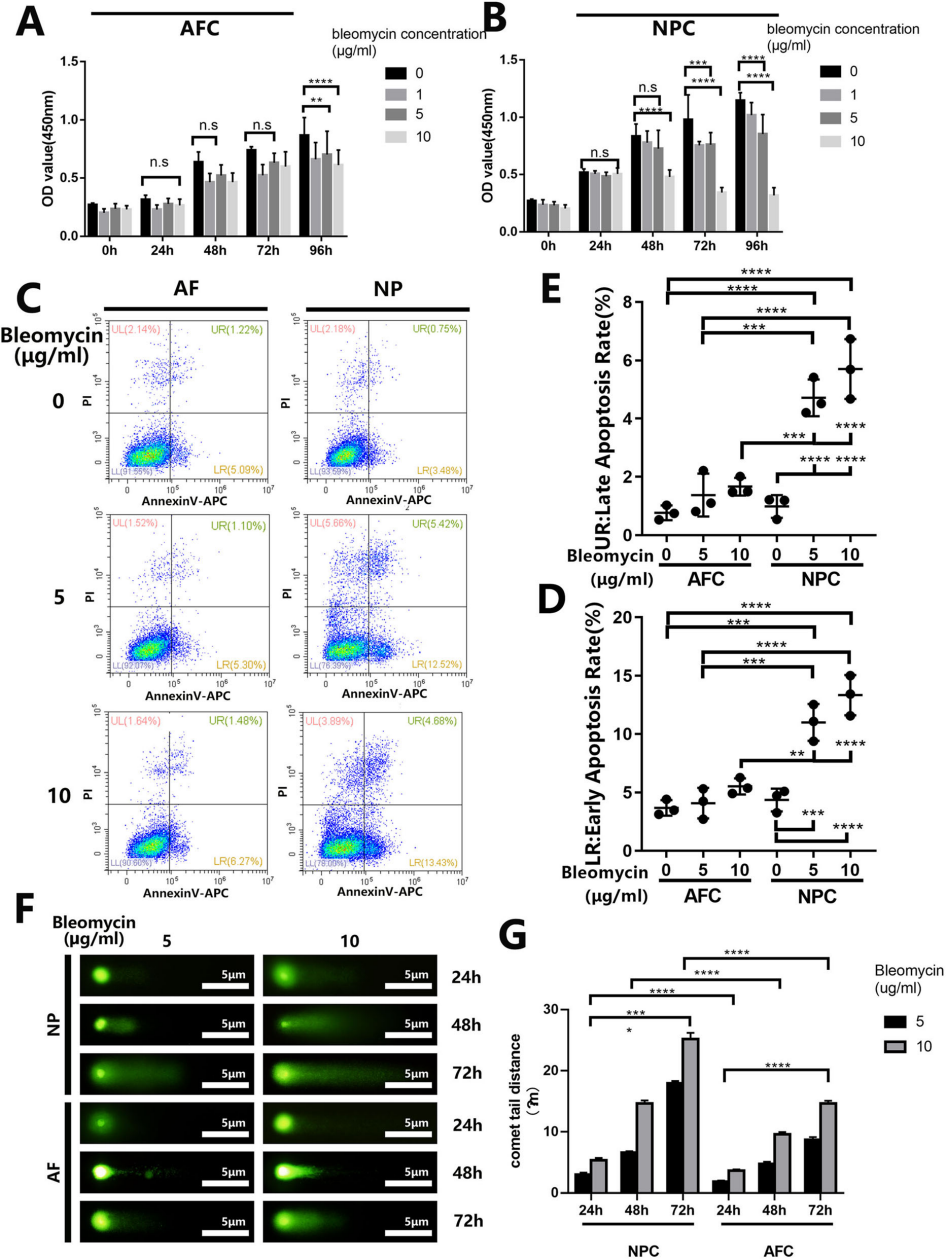
Bleomycin shows little cytotoxicity in AF cells in vitro. **a**, **b** Cell Count Kit-8 test of AF cells and NP cells were stimulated with bleomycin in different concentrations of 0, 1, 5, and 10 μg/ml and different time period ranged from 0 to 96 h. **c** AF cells and NP cells were treated with bleomycin in a concentration of 0, 5, and 10 μg/ml and stained with Annexin V and PI, then subjected to flow cytometric analysis. **d**, **e** Quantification of early and late apoptotic cells rate using Graphpad8.0 by ordinary one-way ANOVA test. **f** AF cells and NP cells were treated with bleomycin in a concentration of 5 and 10 μg/ml then stained with Comet assay kit. **g** The degree of DNA damage was measured by comet tail distance using Graphpad8.0 by ordinary one-way ANOVA test. All data are presented as mean ± sd. from three experiments. *P < 0.05, **P < 0.01, ***P < 0.001, and ****P < 0.0001

Our previous research using a cell-based approach has shown that induction of reparative fibrosis may offer beneficial effects against the progression of disc degeneration [17], and this process is associated with the TGFβ signaling pathway. Considering bleomycin is an effective trigger to induce the upregulation of TGFβ in lung epithelium cells [24] and has been used in clinical situation before, we tried to use bleomycin combined with the multipotentiality of BMCSs to induce their fibrotic differentiation. In our research, bleomycin could efficiently promote the migration of BMSCs in wound healing assay and transwell test (Fig. 2a, b). Then, we showed that the cell viability of BMSCs was minimally deteriorated by the bleomycin in a concentration of 5 and 10μg/ml with a duration of 24 h and 72 h (Fig. 2c), plus, as shown in the Suppl. Figure 1A and B, although the apoptosis rate of BMSCs increased (from 1.56 ± 0.1418%, *n* = 3 to 3.19 ± 0.1601%, n = 3), it is significantly lower than the NP cells (10.99 ± 0.9104%, *n* = 3, Fig. 3d, e). This enhancement in the ability of migration may be the consequence of changes that happened in the molecular level, including the upregulation of TGFβR1 and TGFβ gene, as well as the expression of pro-fibrotic marker genes Col1a1, FSP1, and Fn1(Fig. 2f). Moreover, in the western blotting test, we detected the slight elevation of TGFβR1, Col1a1, and FSP1 with the stimulation of bleomycin (Fig. 2d). Immunofluorescence staining was carried out, and we got the same result that TGFβ and Col1a1 increased with the bleomycin (Fig. 2e). To further confirm our hypothesis, we use LY363947 (10 μM) [25], a TGFβR1 kinase activity inhibitor, to treat BMSCs with bleomycin and found that it markedly inhibited AF cell migration in both the scratch-wound healing and the transwell migration assay (Fig. 2a, b), and it could also mitigate the upregulation of these pro-fibrotic marker genes and proteins mentioned above (Fig. 2e, f).

### Bleomycin shows less cytotoxicity in AF cells and BMSCs in vitro

Bleomycin is a chemotherapeutic cytotoxic compound that inhibits DNA metabolism and causes free radical-based DNA damage. So, we assessed the effects of bleomycin in the intervertebral disc. We first examined the cellular effects of bleomycin on cells of the intervertebral discs particularly AF and NP cells. As shown in Fig. 3a, b, bleomycin exerted no cytotoxic effects on AF and NP cells following 24 h treatment. For AF cells, a trend of decrease in cell viability was observed at all doses of bleomycin when cells were treated for 48 and 72 h (the decreased viability rate of AF cells in a concentration of 5 and 10 μg/ml for 48, 72, and 96 h are 3% and 3%, − 5%, and 5%, 9%, and 10%), but the cytotoxic effect was only statistically significant in 96 h (Fig. 3a). On the other hand, a significant reduction in cell viability was observed in NP cells treated with 10 μg/ml of bleomycin for 48, 72, and 96 h and with 5 μg/ml of bleomycin at 96 h. (Fig. 3b).

Next, we employed flow cytometry to examine whether the cytotoxic effects of bleomycin on NP cell viability were due to the induction of apoptosis. As shown in Fig. 3c, a greater percentage of NP cells underwent cell death (necrotic and apoptotic) following 72 h of bleomycin treatment (at both 5 and 10 μg/ml) when compared with untreated controls as well as with AF cells. Both early and late apoptotic rates in NP cells were elevated significantly and dose-dependently (Fig. 3d, e). On the other hand, the percentage of cells that underwent apoptosis (Fig. 3d, e) in AF cells following bleomycin treatment was similar to the untreated controls. Consistent with the elevated apoptotic tendencies, NP cells treated with bleomycin were found to exhibit reduced expression of Cyclin D1, a cell cycle regulatory protein, and concomitant increase in the levels of cleaved Caspase 3, a crucial executor caspase in the apoptotic signaling cascade (Suppl. Figure 2B).

In the single cell’s level, single-cell gel electrophoresis or comet analysis was carried out. Figure 3f, g and Suppl. Figure 1C and D showed that bleomycin dose-dependently induces significant DNA strand breaks and formation of markedly long comet tails in NP cells, evidenced by fluorescence intensity of DNA in the head and tail of the comet. Although DNA breaks and comet tails were also observed in bleomycin treated AF cells and BMSCs (Fig. 3f, Suppl. Figure 1C), the degree of DNA damage was significantly less than in NP cells both in a dose- and time-dependent manner (Fig. 3g and Suppl. Figure 1D). Taken together, our results show that NP cells are more susceptible to the cytotoxic and DNA damaging effects of bleomycin than AF cells.

To further evaluate the side effect of bleomycin, senescence and cell cycle analyses were carried out on BMSCs and NP cells. For NP cells, bleomycin leads to the cell senescence whatever in the short-term treatment (once for 3 days) or the long-term treatment (three times and every stimulation lasts 3 days). On the contrary with the short-term treatment of the bleomycin, the β-gal stained BMSCs had no change unless the time period increased to 9 days (Suppl. Figure 4A and B). From the perspective of lifespan, the cell cycles of both cells were analyzed. As shown in the Suppl. Figure 4C and D, the cell distribution changed a lot in NP cells with an increased G1 phase and a decreased S phase, but for BMSCs, the distribution of G1 phase, S phase, and the G2 phase was quite the same with bleomycin. Moreover, in the PCR test, there is an increase in the gene expression like P21, CCND2, and CDK6, with a decreased CDK1, CDK2, CCNG2, and CDK4 without change. For western blot assay, there are no changes in P53, P21, and cleaved PARP expression unless the decrease of full-length PARP (Suppl. Figure 5A and B).

### Bleomycin promotes AF cell migration in vitro via TGFβ-TGFβR1 signaling

**Fig. 4:**
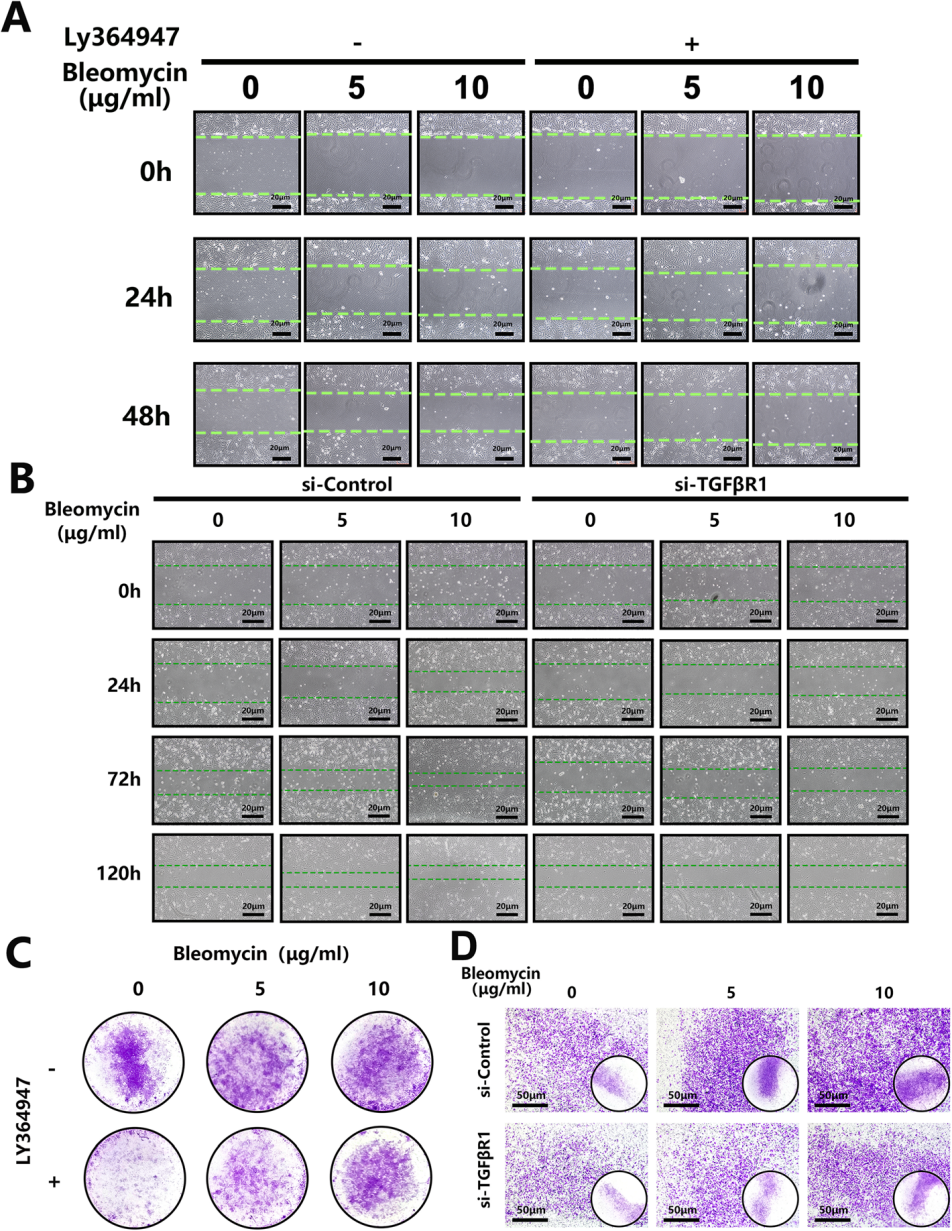
Bleomycin promotes annulus fibrosus cells’ migration in vitro. **a**, **c** AF cells were pretreated with TGFβR1 inhibitor LY364947(10 μM) or DMSO and stimulated with bleomycin in a concentration of 0, 5, and 10 μg/ml, then cells were cultured for 48 h and observed in a checkpoint of 0, 24 h and 48 h to calculate the migration rate by wound healing assay or cultured for 24 h to do the transwell test. **b**, **d** Normal and TGFβR1 knocked-down AF cells were stimulated with bleomycin in a concentration of 0, 5, and 10 μg/ml, then cells were cultured for 48 h and observed in a checkpoint of 0, 24, and 48 h to calculate the migration rate by wound healing assay or cultured for 24 h to do the transwell test

Considering the AF cells are fibroblast-like cells and the TGFβ signaling pathway functioned in the fibroblast cells’ migration [26], we detected the collective migratory behavior of AF cells using the in vitro scratch-wound healing assay. When compared to untreated controls, bleomycin treatment dose-dependently enhanced AF cell migration and scratch-wound closure at 24 and 48 h after the creation of the scratch wound creation (Fig. 4a and Suppl. Figure 3A). To better mimic the in vivo behavior of individual AF cells, the transwell directional migration assay was performed. As shown in Fig. 4c, AF cells that migrated through the transwell inserts were stained with crystal violet. Furthermore, consistent with the scratch-wound healing migration assay, bleomycin treatment significantly increased the number of AF cells that migrated through the transwell insert (Suppl. Figure 3B). This suggests that activated TGFβ-TGFβR1 signaling is involved in mediating the effects of bleomycin on AF cells. To further confirm that this was the case, TGFβR1 gene silencing in AF cells was carried out followed by bleomycin treatment and migration assays. Consistent with the inhibitory effects of LY363947, the knockdown of TGFβR1 significantly attenuated bleomycin-induced AF cell migration in both the scratch-wound healing and transwell migration assays (Fig. 4b, d; and Suppl. Figure 3C and D).

### Bleomycin activates the TGFβ-TGFβR1-SMAD2/3 signaling pathway in AF cells

**Fig. 5:**
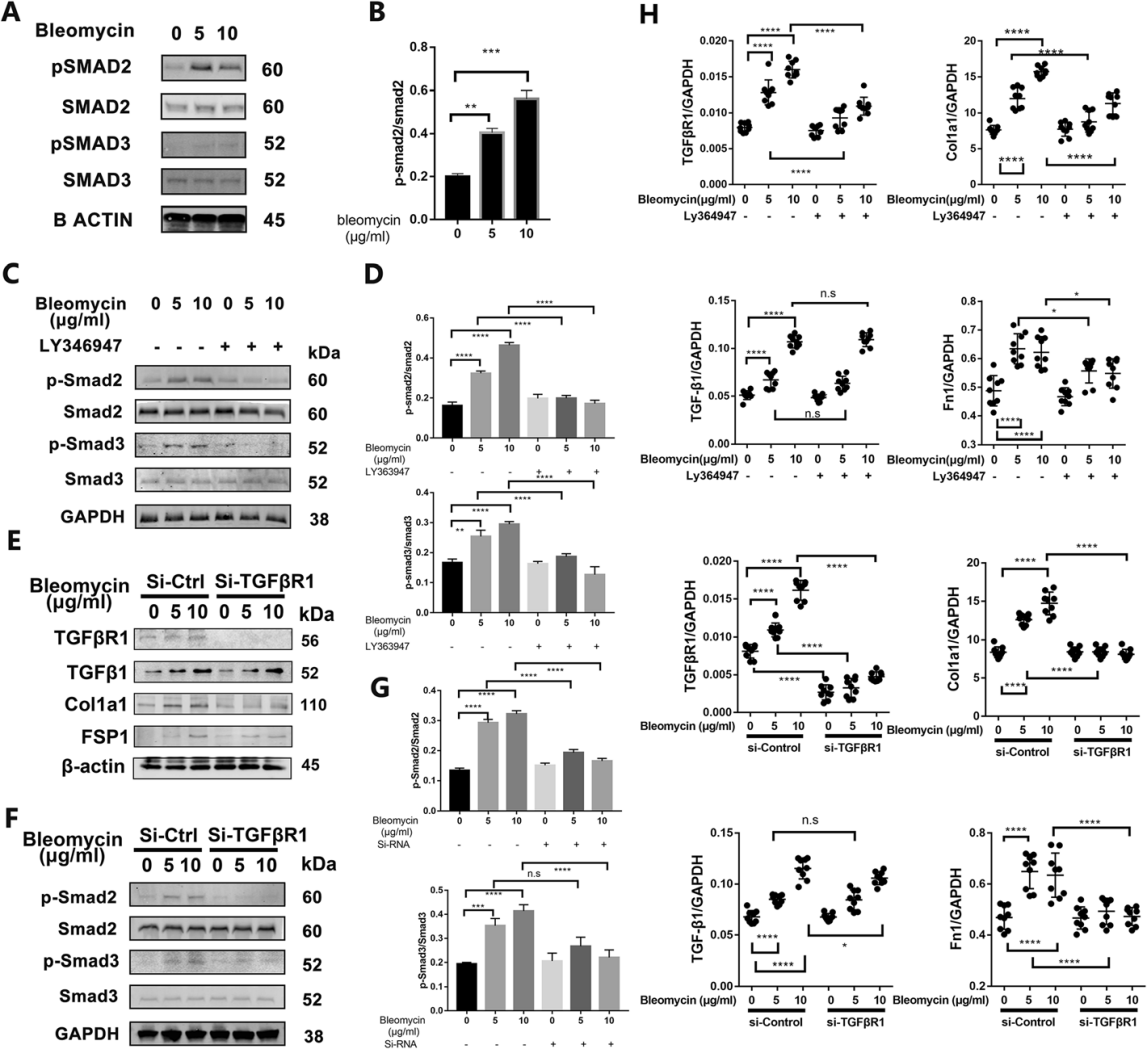
Bleomycin activated the TGF-beta signaling pathway in AF cells. **a**–**d** Western blot analysis of phospho-Smad2, phospho-Smad3, Smad2, and Smad3 in AF cells with bleomycin of 0, 5, and 10 μg/ml pretreated with LY346947 or not and quantification of grayscale value. **e** Western blot analysis of TGFβ, TGFβR1, FSP1, and Col1a1 in AF cells uses β-actin as a reference. **f**, **g** Western blot analysis of phospho-Smad2, phospho-Smad3, Smad2, and Smad3 in AF cells with or without TGFβR1 knocked-down stimulated by bleomycin and quantification of grayscale value. **h** Q-PCR analysis of the relative mRNA expression levels of TGFβ, TGFβR1, Fn1, and Col1a1 in AF cells with bleomycin or/and LY364947, with or without TGFβR1 knocked-down stimulated by bleomycin. All qPCR data are calculated with Graphpad8.0 by ordinary one-way ANOVA test and presented as mean ± sd. from three experiments. *P < 0.05, **P < 0.01, ***P < 0.001, and ****P < 0.0001

To further decipher the molecular mechanism for the effects of bleomycin on AF cells, biochemical analyses of gene and protein expression were carried out. As shown in Fig. 5h and Suppl. Figure 6A, bleomycin treatment induced the upregulation TGFβR1, TGFβR2, and TGFβ gene expression, as well as the expression of pro-fibrotic marker genes Col1a1, Col3a1, and Fn1, but no change in the TGFβR3 gene expressions was observed. Pharmacological treatment with LY363947 inhibited bleomycin-induced upregulation of TGFβR1, Col1a1, Col3a1, and Fn1 gene expression but had little effect on TGFβ and TGFβR2. Silencing of TGFβR1, of which the knock-down efficiency is approximately 66.9%, significantly inhibited these changes better than LY363947 treatment but still could not inhibit TGFβ and TGFβR2 neither (Fig. 5h and Suppl. Figure 6B). Nonetheless, these results indicate that bleomycin induced AF cells to adopt a stronger fibrotic phenotype.

Immunoblot analyses were then carried out to examine the activation state of TGFβ-TGFβR1 signaling following bleomycin treatment. In our immunoblot assays, we showed that bleomycin treatment induced the expression of TGFβ and TGFβR1(Fig. 5e), and the fibroblast marker FSP1 and pro-fibrotic protein Col1a1 were similarly induced. On the other hand, silencing of TGFβR1 abolished the bleomycin-induced protein expression of Col1a1 and FSP1 (Fig. 5e). Furthermore, considering the downstream activation of SMAD2/3 transcription factors is one of the important drivers of fibrosis, phosphorylation of SMAD2 and SMAD3 was analyzed following bleomycin treatment (Fig. 5a, b). However, pre-treatment of AF cells with LY363947 abolished the bleomycin-induced activation of TGFβ-TGFβR1-SMAD2/3 signaling (Fig. 5c, d). Similar inhibitory effects against bleomycin-induced activation of TGFβ-TGFβR1-SMAD2/3 signaling were seen following TGFβR1 gene silencing (Fig. 5f, g).

### Bleomycin induces fibrosis in degenerative intervertebral discs and maintains disc height in vivo

**Fig. 6:**
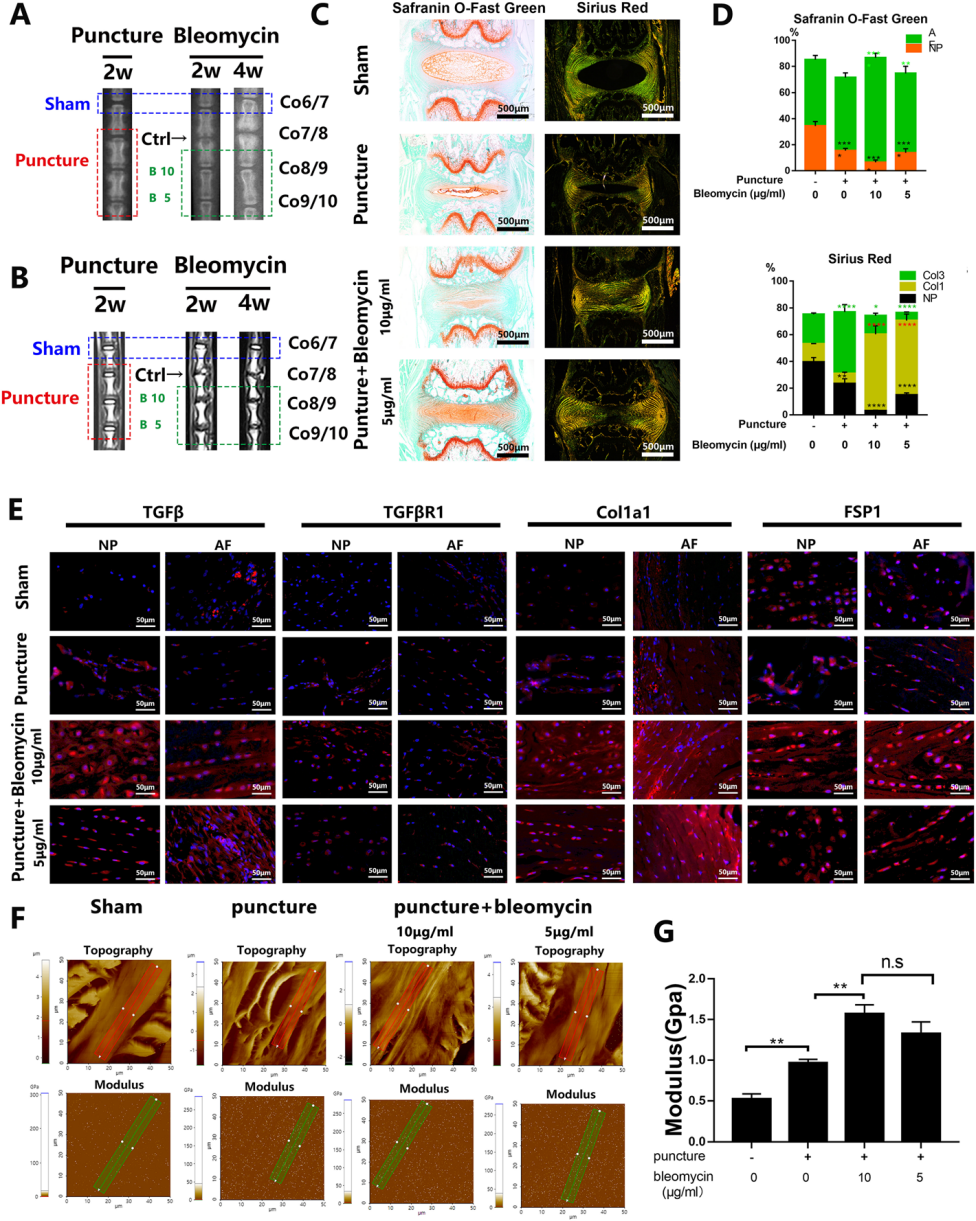
Bleomycin induces degenerated disc fibrosis without height loss in rats. All rats were punctured at Co7/8, Co8/9, and Co9/10, then after 2 weeks, Co8/9 and Co9/10 were rescued by bleomycin in a concentration of 5 and 10 μg/ml. **a** X-ray tests of rats at a prone posture, and then the pictures were cropped focused on the area of operation. **b** MRI tests of rats at a supine posture and the T2 signal pictures were chosen. **c** The tails for operation (Co6/7, Co7/8, Co8/9, and Co9/10) were dissected and used to make paraffin section with subsequent Safranin O-Fast Green stain and Sirius Red stain. **d** Proportion quantification of the area represent the AF region, fibrosis NP region in Safranin O-Fast Green stain, Col1a1, Col3a1, and fibrosis NP region in Sirius Red stain using IPP6.0 and calculated by Graphpad8.0 with ordinary one-way ANOVA test. **e** Immunofluorescence assay of TGFβ, TGFβR1, FSP1, and Col1a1 in the fibrosis NP region and AF region described in **c**. **f**, **g** Atomic force microscopic of the fibrosis AF region in the paraffin section and Quantification of Young’s modulus using the XEI of Park System. All data are presented as mean ± sd. from three experiments. *P < 0.05, **P < <0.01, ***P < 0.001, and ****P < 0.0001

Using a needle puncture model of intervertebral disc degeneration in rats, we validated the potential use of bleomycin to induce reparative fibrosis for the maintenance of disc height. Disc degeneration was allowed to develop for 2 weeks after the needle puncture prior to administration of bleomycin therapy for further 2 and 4 weeks (Suppl. Figure 6C, E and F). X-ray and MRI imaging were carried out to evaluate the effects of bleomycin therapy (Fig. 6a, b). X-ray radiographs show that the intervertebral dics that underwent needle puncture without subsequent bleomycin treatment (Co7/8) showed progressive disc degeneration including height loss and disc collapse by the end of the experimental procedure (Fig. 6a). MRI scans further show disc degeneration with a progressive increase in gray levels (loss of T2 signal intensity) in the needle puncture only intervertebral discs (Fig. 6b). In contrast, intervertebral discs that received bleomycin therapy (Co8/9, 10 μg/ml; Co9/10, 5 μg/ml) exhibit little degenerative features and maintenance of disc height despite the progressive loss of T2 signal intensity (Fig. 6a, b). No degeneration of the intervertebral discs was observed in the sham group.

Histological examinations were then carried out to further assess the microstructural changes following bleomycin therapy (Fig. 6c, d). In both SOFG and Sirius Red-stained intervertebral disc sections, AF and NP boundaries are clearly demarcated with no signs of disc degeneration (Fig. 5c). The lack of collagen content was observed in the NP, whereas AF exhibits abundant type 3 and type 1 collagen (Fig. 6c, d). Following the needle puncture, we can see the loss of disc height, collapse of the intervertebral discs, increased type 3 collagen content in the NP indicating the onset of fibrosis (Fig. 6c, d). However, no fibrosis in the remaining NP was observed (Fig. 6c). In stark contrast, the boundaries between the AF and NP were not discernible in the bleomycin therapy groups, with the NP completely filled with concentric lamellar type I collagen 1 (Fig. 6c, d) Interestingly, to further investigated the cell distribution in the discs, we used KRT18 to stain the NP cells and S100A9 to stain the AF cells and got the result that the ratio of the NP cells and AF cells reduced with bleomycin stimulation (Suppl. Figure 7). The formation of the fibrous tissue and the increased fibroblast-like AF cells resulted in the maintenance of the intervertebral disc height (Fig. 6c). Immunofluorescence staining was further carried out to assess the expression of pro-fibrotic markers in the intervertebral discs. As shown in Fig. 6e, untreated degenerative discs exhibited slightly elevated expression of TGFβ, TGFβR1, Col1a1, and FSP and decreased Col2a1 in the NP when compared to the sham controls. Consistent with in vitro effects, bleomycin administration significantly upregulated the expression of TGFβ, TGFβR1, Col1a1, and FSP in the AF and NP, but for Col2a1 (Suppl. Figure 5C), it showed a bi-directional effect, which increased in the NP region and deceased in the AF region.

Atomic force microscopy (AFM) was subsequently carried out to evaluate the structural properties and stress tolerance, which was described by Young’s modulus (a measure of ability to withstand elastic/recoverable deformation under lengthwise tension or compression; a measure of stiffness), adhesion force, deformation, and the topography correlated with the displacement curve (Suppl. Figure 8B and 8D). With puncture, Young’s modulus increased in the AF region, but after bleomycin injection, Young’s modulus was even significantly higher (Fig. 5f and 6g). Conversely, we observed elevation of the Young modulus in the NP region while puncture, but there was relative recovery after bleomycin rescue (Suppl. Figure 8A and C), meaning the NP region could maintain the nature biomechanical status with bleomycin. Collectively, these results provide evidence that bleomycin therapy can accelerate the IVD fibrosis and strengthen the discs’ anti-stress ability without loss of the disc height.

## Discussion

The incidence of intervertebral disc degeneration is increasing at an alarming rate in our modern society, and the height loss that happened during degeneration is the major cause of neurological symptoms. Following the height loss was the change of biomechanical characteristic [27] and the possible compression of the nerve root in intervertebral foramina [28, 29], so aiming at maintaining even restoring the height is the effective treatment to solve the problem. However, a lack of effective early and mid-term treatment options to maintain the height is available with most end-stage diseases treated surgically via discectomy and interbody spinal fusion. Some previous research tried to use the regeneration methods to maintain the disc height, like A bio-scaffold composed of decellularized nucleus pulposus and native nucleus pulposus [30] or just an in situ gelling alginate hydrogel [31] alone, and in our research, we offered a new hypothesis that the reparative fibrosis model combined with BMSCs and bleomycin might be an alternative choice to treat IVDD.

**Fig. 7:**
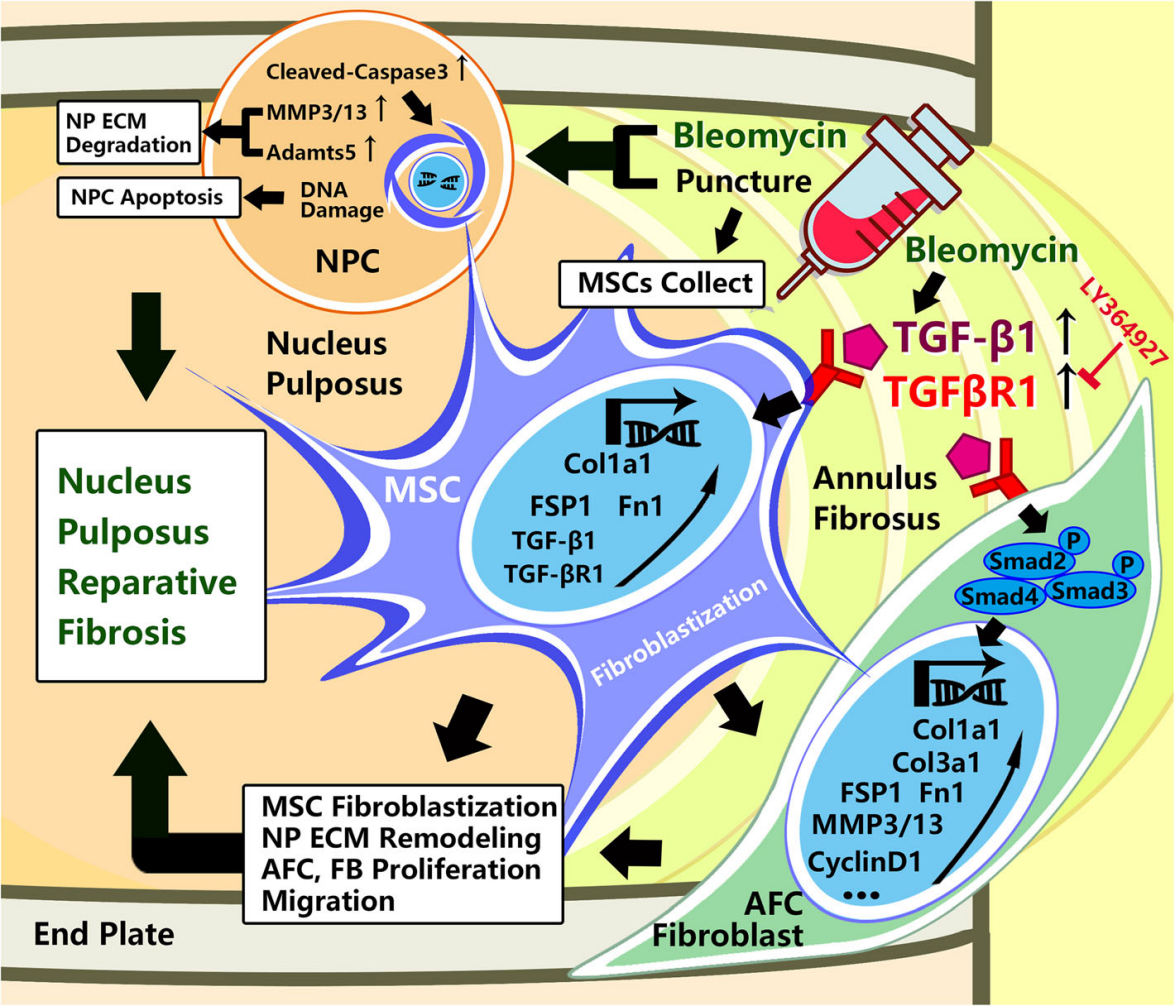
Regulatory function of bleomycin in the fibroblastization of BMSCs in the microenvironment of the nucleus pulposus. Bleomycin could induce the fibroblastization of BMSCs; it promotes the fibroblastic transformation of AF cells by increasing the expression of TGFβ and TGFβR1, which activates the TGFβ/Smad2,3 signaling pathway, and finally by increasing the proliferation and migration of these cells. On the other hand, it could stimulate the NP cells to secrete MMP3 and MMP13 to remodel the ECM in the NP that is beneficial for the migration of fibroblastic cells. Locally, injection of bleomycin would increase the Col1a1 and Col3a1 then finally lead to accelerated reparative fibrosis

Disc fibrosis is in fact a compensatory process during disc degeneration, but the cells that functioned during this process still have been debated during the past few years (Fig. 7). Some researchers have found that there are some proliferated cells which shared the same markers with MSCs in the human degenerated IVDs [32, 33]; these cells are distributed mainly in the endplate or the outer AF region [34], and their migration rate and endogenous repair mechanism still need to be determined [16, 35]. From our previous observations in patients, we observed fibrotic changes in the acute phase of lumbar disc herniation as well as fibrosis of the intervertebral disc after percutaneous endoscopic lumbar discectomy. Such changes could represent an initial compensatory protective mechanism to maintain the height of the intervertebral disc and spine, which the MSCs may play an important and initial role during this process. Hence, from our perspective, we advocate to activate the MSCs and fibroblast-like cells, promote its ability of migration and collagen deposition aiming at finally alternate the declining NP tissues and successfully maintain even restore the disc height. The induction of reparative and fast fibrosis in degenerative intervertebral discs could offer a way for tissue repair and spine stabilization. We and others have previously shown that reparative fibrosis defined here as the formation of organized scar tissue necessary to mechanically stabilize the degenerative intervertebral disc has potential beneficial effects against the height loss during intervertebral disc degeneration [17, 36]. The use of dermal fibroblast-based cell therapy was found to induce degenerative intervertebral disc fibrosis, prevent the loss of disc height and disc collapse, and maintain the biomechanical properties of the spine [37, 38].

In this study, we use bleomycin, a chemotherapeutic antibiotic and pro-fibrotic agent, to induce intervertebral disc fibrosis following needle puncture-induced disc degeneration in rats. X-ray radiographs, MRI imaging, and AFM analysis found that the pharmacological induction of fibrosis of the discs by bleomycin could also maintain intervertebral disc height. BMSCs were complicated during their whole lifespan; our in vitro cell-based assays showed that bleomycin would not lead to a significant change in their cell cycle and apoptosis rate in short-term use, which preserved the possibility of future clinical usage. Moreover, the short-term bleomycin could induce BMSCs towards a stronger fibroblast phenotype, although with long-term usage it might cause the cells to undergo aging (in some studies, the senescence of BMSCs or other cells was found to be associated with the transition into fibrotic phenotype [39, 40]), and for fibroblastic-like cell AF cells, it could promote the migration and collagen deposition via the TGFβ-TGFβR1-SMAD2/3 signaling pathway. Bleomycin could upregulate the MMP3 and MMP13 to remodel the ECM (Suppl. Figure 3E), which might help the AF cells to migrate. Despite the structural and compositional differences between healthy NP tissue and fibrotic tissue, the ability of the fibrotic tissue in the NP to maintain the intervertebral disc height may help offset pressure on the surrounding tissues and restore some degree of biomechanical stability to the degenerative intervertebral discs.

Mechanistically, we showed using both cellular and molecular-based assays that the TGFβ-TGFβR1-SMAD2/3 is at least involved in the transition of AF cells and BMSCs towards a stronger pro-fibrotic phenotype. This was supported by immunofluorescence staining of intervertebral disc sections from our in vivo intervertebral disc degeneration model which show elevated expression of TGFβ, FSP1, and type I collagen in the AF. TGFβ is a potent pro-fibrotic cytokine central to the development of tissue fibrosis. TGFβ modulates fibroblast proliferation and migration and stimulates ECM production and collagen deposition [41]. Binding of TGFβ to its target receptor (TGFβRI/ALK5 or TGFβRII) on target cells recruits and activates the receptor-regulated effector proteins (R-SMADs), particularly SMAD2 and SMAD3 through direct C-terminal phosphorylation of an SSXS motif. The activated (phosphorylated) SMAD2/3 form trimeric complexes with SMAD4 to translocate into the nucleus to initiate the activation or repression of target genes in cooperation with other transcription factors [42, 43]. The TGFβ-SMAD2/3 signaling pathway has been shown to play a key role in the fibrotic process, and the dysregulation of TGFβ-SMAD2/3 leads to pathological tissue fibrosis of the lung, liver, and kidney [44–47]. Acting through the TGFβ-TGFβR1-SMAD2/3 axis, we found that bleomycin promoted migration of AF cells and BMSCs, and also upregulated the expression of key ECM genes such as type 1 collagen as well as type 3 collagen, FN1 in AF cells [48–50]. Pharmacological inhibition of TGFβ signaling with LY346947, a TGFβR1 kinase inhibitor, or with TGFβR1 gene silencing, significantly attenuated the bleomycin-induced cell migration.

## Conclusions

Our pilot study has demonstrated the potential of bleomycin-induced fibrosis in the management of intervertebral disc degeneration. Bleomycin-induced fibrosis not only maintains intervertebral disc height but also improves the stress tolerance of the degenerative disc. Certainly, more detailed investigations are needed to warrant the potential usefulness of bleomycin therapy for the treatment of intervertebral disc degeneration in clinical practice.

## Supplementary Information

**Additional file 1: Sup Figure 1.** (a) BMSCs were treated with Bleomycin in a concentration of 0, 5 μg/ml and stained with Annexin V and PI, then subjected to flow cytometric analysis. (b) Quantification of early and late apoptotic cells rate using Graphpad8.0 by ordinary one-way ANOVA test. (c) BMSCs were treated with Bleomycin in a concentration of 0, 5 μg/ml then stained with Comet assay kit. (g) The degree of DNA damage was measured by comet tail distance using Graphpad8.0 by ordinary one-way ANOVA test. All data are presented as mean ±sd. from three experiments. *P<0.05, **P<0.01, ***P<0.001 and ****P<0.0001.

**Additional file 2: Sup Figure 2.** (a) Cell death rate calculated by Graphpad8.0 by ordinary one-way ANOVA test of cells described at Fig. 2d. (b) Western blot analysis of CyclinD1 and Cleaved Caspase3 in AF and NP cells stimulated with bleomycin of 0, 5 and 10 μg/ml. (c) Q-PCR analysis of the relative mRNA expression levels of TGFβ, TGFβR1, Col1a1 and Fn1 in NP cells with Bleomycin. (d) Western blot analysis of phospho-Smad2, phospho-Smad3, Smad2 and Smad3 in NP cells with Bleomycin. All data are presented as mean ±s. d. from three experiments. *P<0.05, **P<0.01, ***P<0.001 and ****P<0.0001.

**Additional file 3: Sup Figure 3.** (a, b, c, d) Quantification of distance in wound healing assay and migration rate of transwell test for cells described at figure 4a, b, c, d. (e) Q-PCR analysis of the relative mRNA expression levels of MMP3, MMP13 and Timp1 in NP cells with Bleomycin and Ly363937 or not. All data are presented as mean ±s. d. from three experiments. *P<0.05, **P<0.01, ***P<0.001 and ****P<0.0001.

**Additional file 4: Sup Figure 4.** (a) NP cells and BMSCs were treated with Bleomycin in a concentration of 5 μg/ml once or three times and stained with β-gal staining buffer. (b) Quantification of the cells percentage stained with β-gal or not of cells in Sup figure 4. (c) NP cells and BMSCs were treated with Bleomycin in a concentration of 5 μg/ml once stained with PI buffer with RNase A, then subjected to flow cytometric analysis. (d) Quantification of the cells distribution by Cell Cycles Simulation in Sup figure 4c. All data are presented as mean ±s. d. from three experiments. *P<0.05, **P<0.01, ***P<0.001 and ****P<0.0001.

**Additional file 5: Sup Figure 5.** (a) Q-PCR analysis of the relative mRNA expression levels of CDK1, CDK2, CDK4, CDK6, CCND2, CCNG2 and P21 in BMSCs with Bleomycin or not. (b) Western blot analysis of the protein expression levels of PARP, cleaved PARP, P21 and P53 in BMSCs with Bleomycin or not. (d) Immunofluorescence assay of Col2a1 in the fibrosis NP region and AF region described in Fig. 6c.

**Additional file 6: Sup Figure 6.** All rats were punctured at Co7/8, and tails of operation (Co6/7, Co7/8) were dissected and used to make paraffin section. (a,b) Q-PCR analysis of the relative mRNA expression levels of TGFβR2, TGFβR3 and Col3a1 in AF cells with Bleomycin or/and LY364947, with or without TGFβR1 knocked-down stimulated by Bleomycin. (c) Immunofluorescence assay of TGFβ, TGFβR1, FSP1 and Col1a1 in the fibrosis NP region and AF region. (d) IOD level of the red region described in a and Fig. 6e were analyzed by IPP.6.0 and subsequently calculated with Graphpad8.0 by ordinary one-way ANOVA. (e) Safranin O-Fast Green stain and Sirius Red stain of the paraffin section. (f) Proportion quantification of area represent AF region, fibrosis NP region in Safranin O-Fast Green stain, Col1a1, Col3a1 and **fibrosis NP region** in Sirius Red stain using IPP6.0 and calculated by Graphpad8.0 by Student-t test. All data are presented as mean ±sd. from three experiments. *P<0.05, **P<0.01, ***P<0.001 and ****P<0.0001.

**Additional file 7 **: **Sup Figure 7.** (a, c) Immunofluorescence assay of KRT18 and S100A4 in the fibrosis NP region and AF region. (b, d) The ratio IOD level between the gree region and the red region described in Sup Figure 7a were analyzed by IPP.6.0 and subsequently calculated with Graphpad8.0 by ordinary one-way ANOVA. All data are presented as mean ±sd. from three experiments. *P<0.05, **P<0.01, ***P<0.001 and ****P<0.0001.

**Additional file 8: Sup Figure 8.** (a, c) Atomic Force Microscopic of fibrosis NP region in the paraffin section mentioned in Figure 6 and Quantification of Young’s Modulus. (b, d) Evaluation of Topography-Displacement, Adhesion Force-Displacement, Young’s Modulus-Displacement and Deformation-Displacement curve. All data are presented as mean ±sd. from three experiments. *P<0.05, **P<0.01, ***P<0.001 and ****P<0.0001.

## Data Availability

All data and materials included in this study are available upon request by contacting the corresponding author.

## Abbreviations

IVDD: Intervertebral disc degeneration
IVD: Intervertebral disc
NP: Nucleus pulposus
AF: Annulus fibrosus
MSCs: Marrow stromal cells
LDH: Lumbar disc herniation
DLS: Degenerative lumbar spondylolisthesis
MRI: Magnetic resonance imaging
AFM: Atomic force microscopy
ECM: Extracellular matrix
