# Supplementary figures and images for: Bleomycin induces fibrotic transformation of bone marrow stromal cells to treat height loss of intervertebral disc through the TGFβR1/Smad2/3 pathway
Source: Stem Cell Res Ther. 2021 Jan 7;12:34. doi: 10.1186/s13287-020-02093-9 (PMC7791639; doi:10.1186/s13287-020-02093-9)

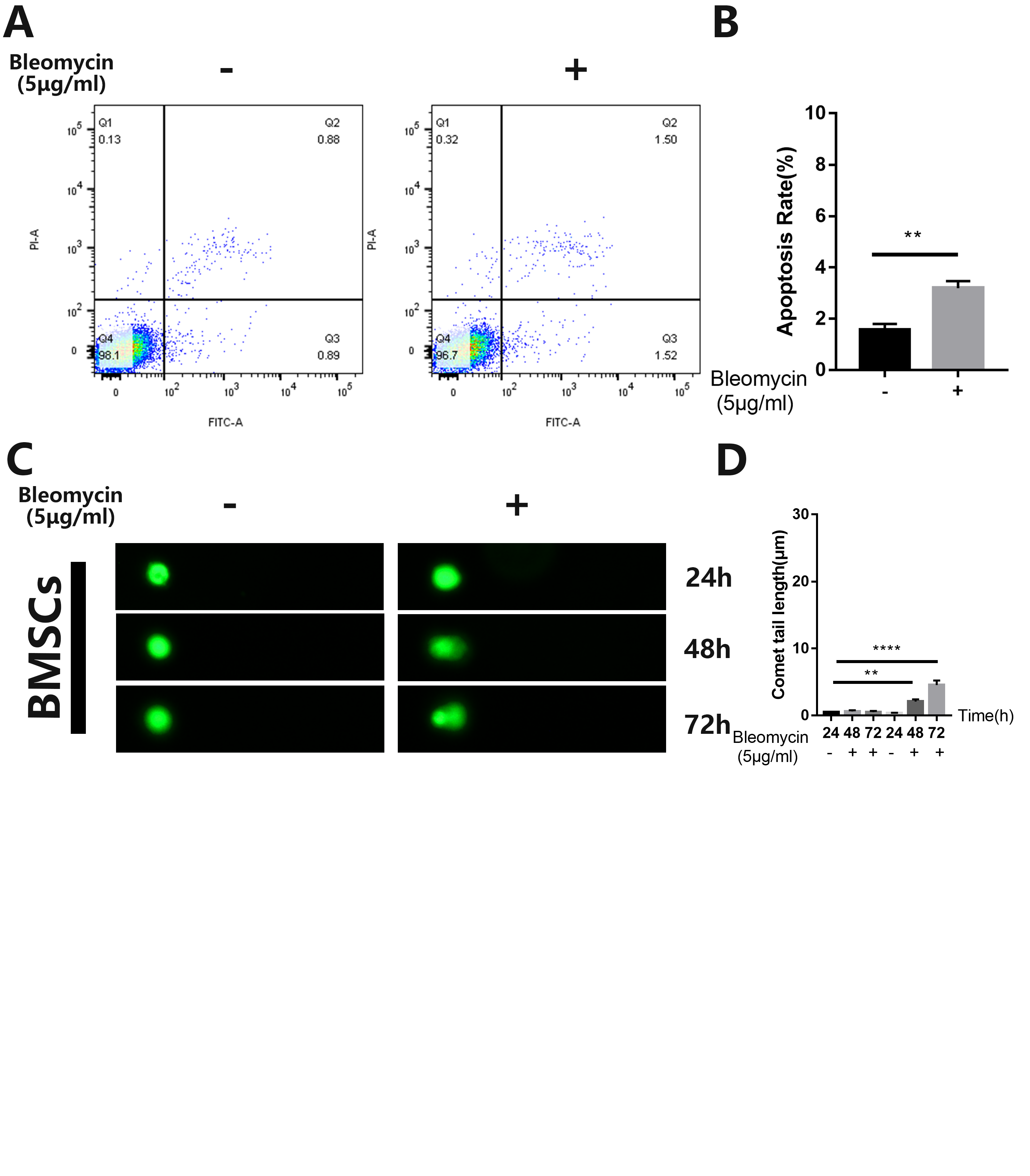

Supplement: Supplementary file 1 — Additional file 1: Sup Figure 1. (a) BMSCs were treated with Bleomycin in a concentration of 0, 5 μg/ml and stained with Annexin V and PI, then subjected to flow cytometric analysis. (b) Quantification of early and late apoptotic cells rate using Graphpad8.0 by ordinary one-way ANOVA test. (c) BMSCs were treated with Bleomycin in a concentration of 0, 5 μg/ml then stained with Comet assay kit. (g) The degree of DNA damage was measured by comet tail distance using Graphpad8.0 by ordinary one-way ANOVA test. All data are presented as mean ±sd. from three experiments. *P<0.05, **P<0.01, ***P<0.001 and ****P<0.0001. [file 13287_2020_2093_MOESM1_ESM.tif]

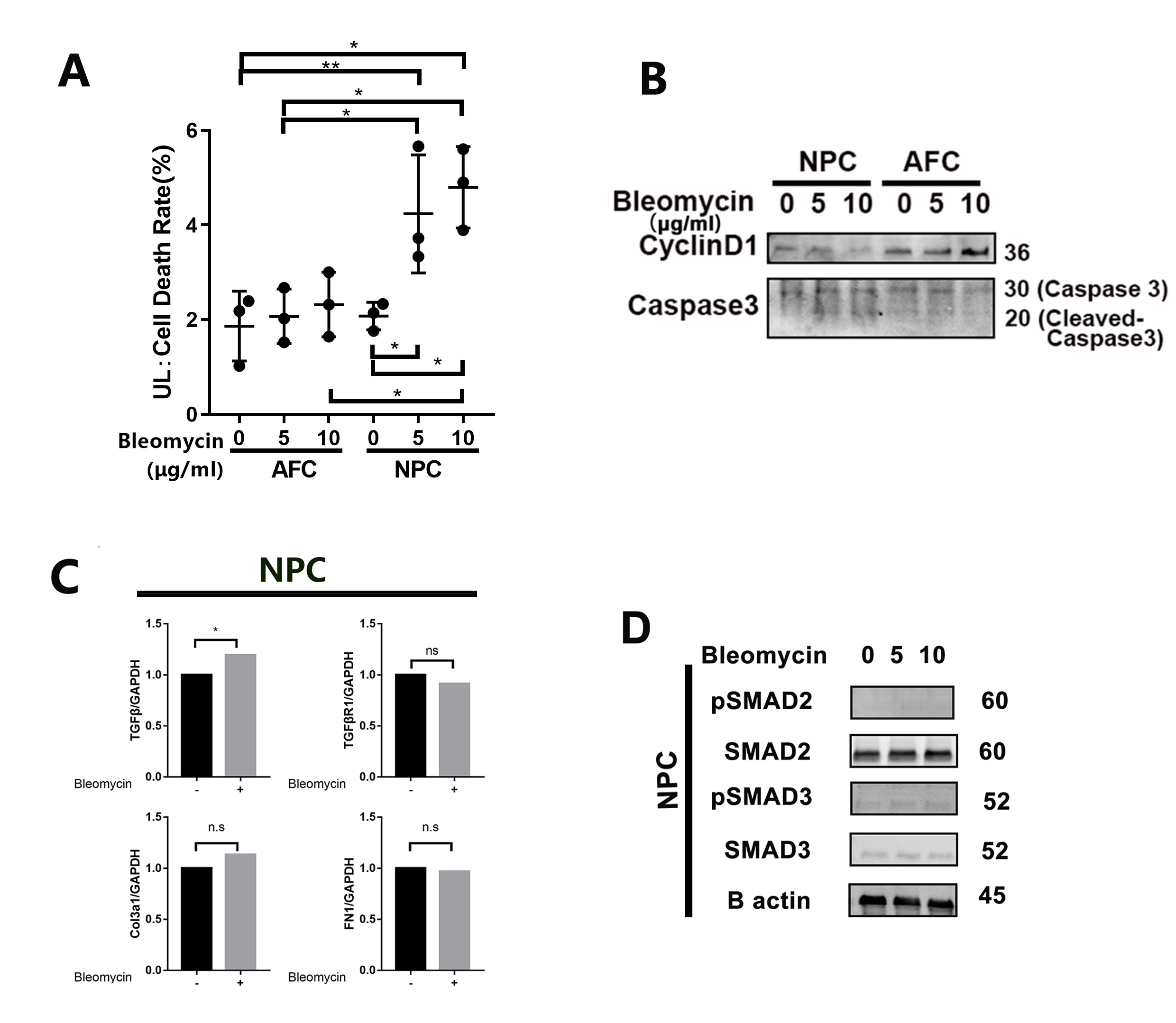

Supplement: Supplementary file 2 — Additional file 2: Sup Figure 2. (a) Cell death rate calculated by Graphpad8.0 by ordinary one-way ANOVA test of cells described at Fig. 2d. (b) Western blot analysis of CyclinD1 and Cleaved Caspase3 in AF and NP cells stimulated with bleomycin of 0, 5 and 10 μg/ml. (c) Q-PCR analysis of the relative mRNA expression levels of TGFβ, TGFβR1, Col1a1 and Fn1 in NP cells with Bleomycin. (d) Western blot analysis of phospho-Smad2, phospho-Smad3, Smad2 and Smad3 in NP cells with Bleomycin. All data are presented as mean ±s. d. from three experiments. *P<0.05, **P<0.01, ***P<0.001 and ****P<0.0001. [file 13287_2020_2093_MOESM2_ESM.tif]

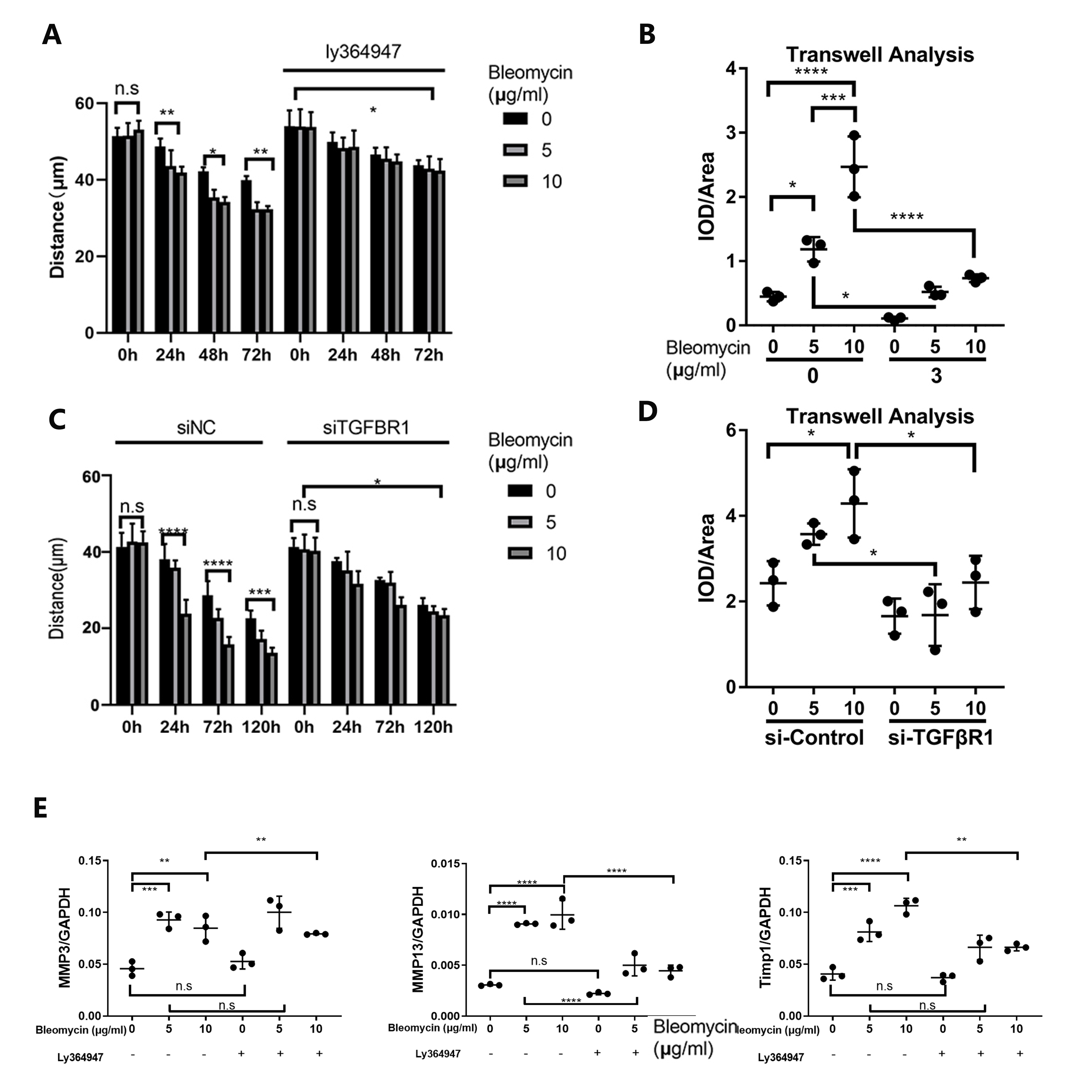

Supplement: Supplementary file 3 — Additional file 3: Sup Figure 3. (a, b, c, d) Quantification of distance in wound healing assay and migration rate of transwell test for cells described at figure 4a, b, c, d. (e) Q-PCR analysis of the relative mRNA expression levels of MMP3, MMP13 and Timp1 in NP cells with Bleomycin and Ly363937 or not. All data are presented as mean ±s. d. from three experiments. *P<0.05, **P<0.01, ***P<0.001 and ****P<0.0001. [file 13287_2020_2093_MOESM3_ESM.tif]

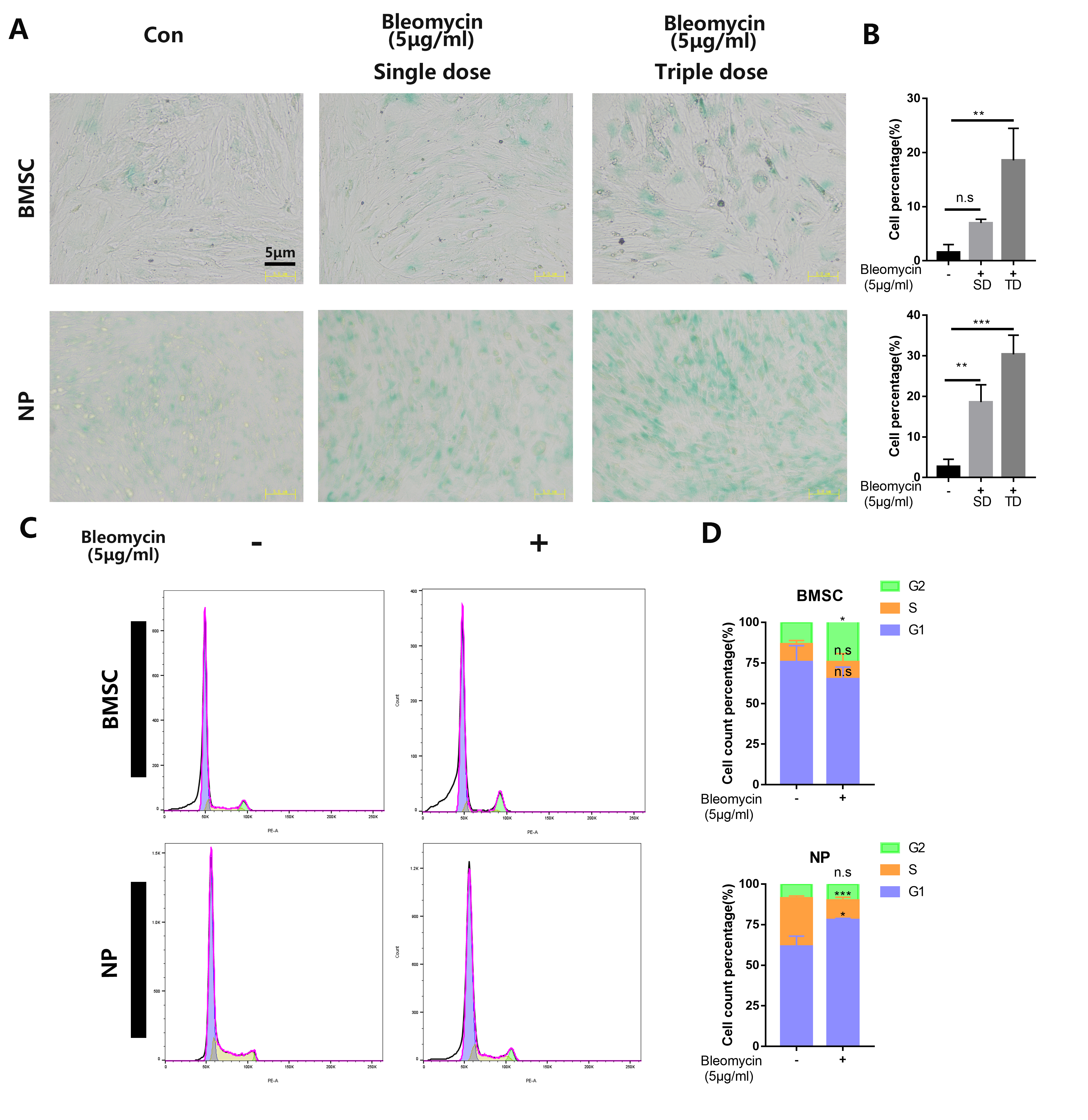

Supplement: Supplementary file 4 — Additional file 4: Sup Figure 4. (a) NP cells and BMSCs were treated with Bleomycin in a concentration of 5 μg/ml once or three times and stained with β-gal staining buffer. (b) Quantification of the cells percentage stained with β-gal or not of cells in Sup figure 4. (c) NP cells and BMSCs were treated with Bleomycin in a concentration of 5 μg/ml once stained with PI buffer with RNase A, then subjected to flow cytometric analysis. (d) Quantification of the cells distribution by Cell Cycles Simulation in Sup figure 4c. All data are presented as mean ±s. d. from three experiments. *P<0.05, **P<0.01, ***P<0.001 and ****P<0.0001. [file 13287_2020_2093_MOESM4_ESM.tif]

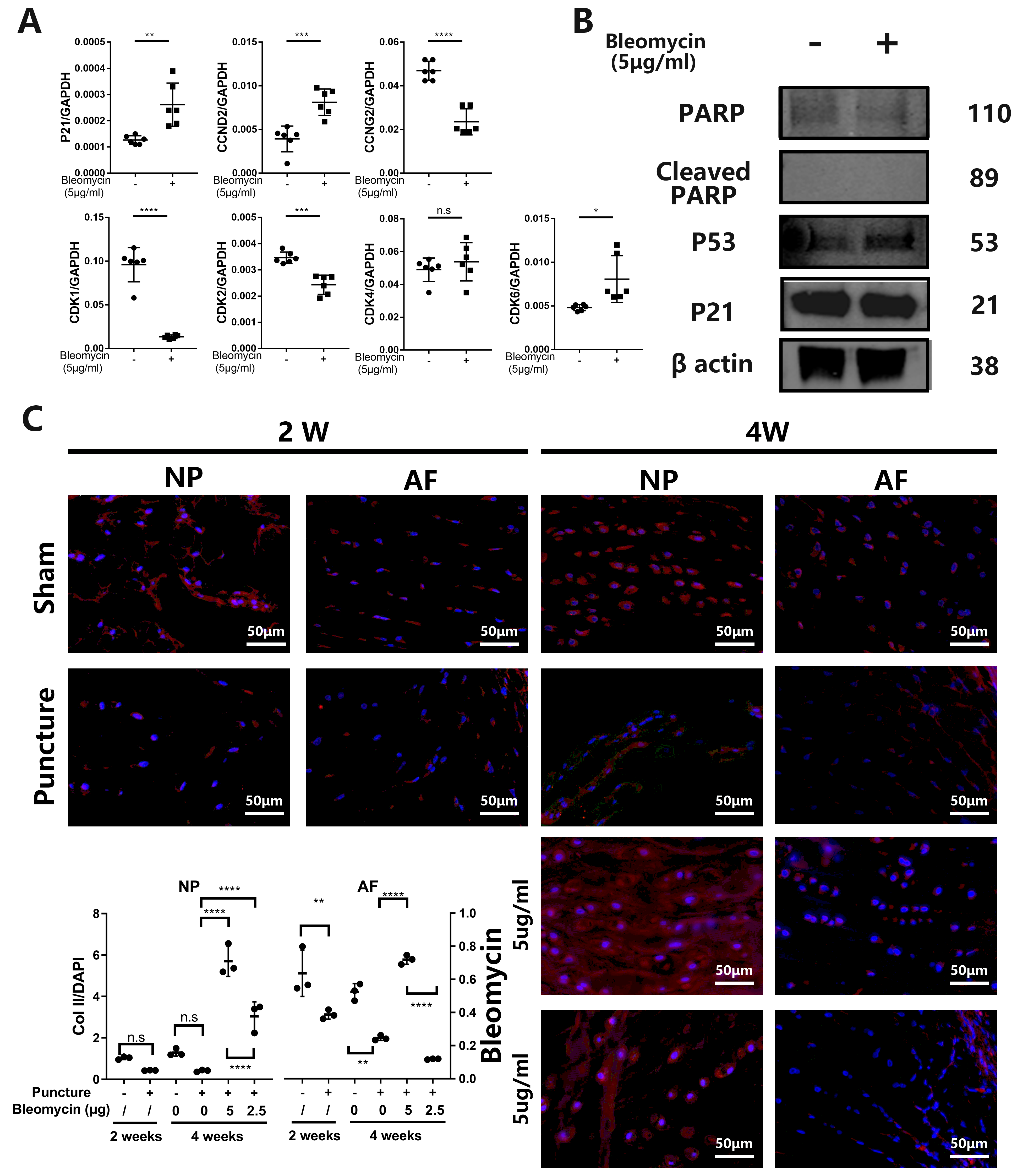

Supplement: Supplementary file 5 — Additional file 5: Sup Figure 5. (a) Q-PCR analysis of the relative mRNA expression levels of CDK1, CDK2, CDK4, CDK6, CCND2, CCNG2 and P21 in BMSCs with Bleomycin or not. (b) Western blot analysis of the protein expression levels of PARP, cleaved PARP, P21 and P53 in BMSCs with Bleomycin or not. (d) Immunofluorescence assay of Col2a1 in the fibrosis NP region and AF region described in Fig. 6c. [file 13287_2020_2093_MOESM5_ESM.tif]

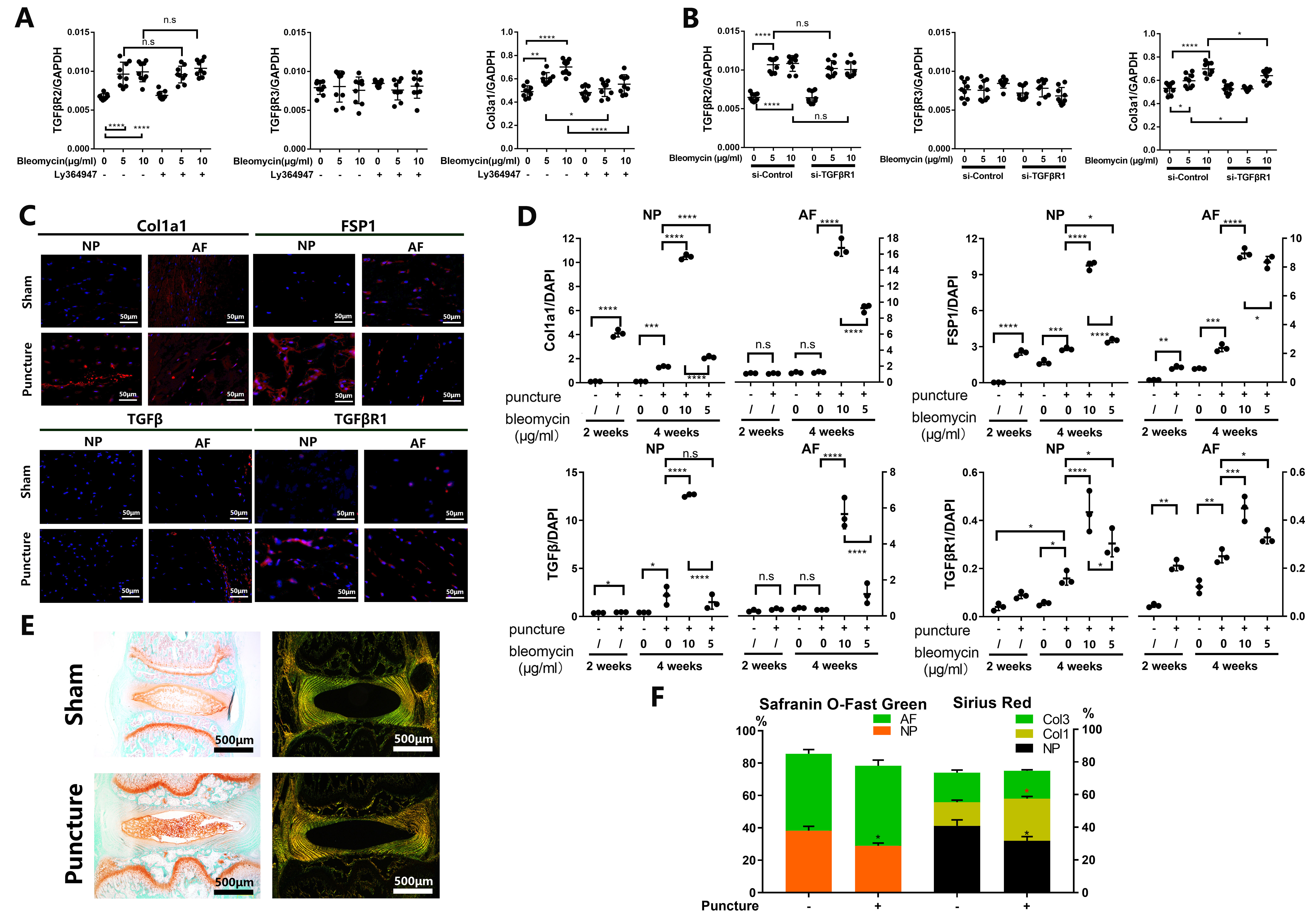

Supplement: Supplementary file 6 — Additional file 6: Sup Figure 6. All rats were punctured at Co7/8, and tails of operation (Co6/7, Co7/8) were dissected and used to make paraffin section. (a,b) Q-PCR analysis of the relative mRNA expression levels of TGFβR2, TGFβR3 and Col3a1 in AF cells with Bleomycin or/and LY364947, with or without TGFβR1 knocked-down stimulated by Bleomycin. (c) Immunofluorescence assay of TGFβ, TGFβR1, FSP1 and Col1a1 in the fibrosis NP region and AF region. (d) IOD level of the red region described in a and Fig. 6e were analyzed by IPP.6.0 and subsequently calculated with Graphpad8.0 by ordinary one-way ANOVA. (e) Safranin O-Fast Green stain and Sirius Red stain of the paraffin section. (f) Proportion quantification of area represent AF region, fibrosis NP region in Safranin O-Fast Green stain, Col1a1, Col3a1 and fibrosis NP region in Sirius Red stain using IPP6.0 and calculated by Graphpad8.0 by Student-t test. All data are presented as mean ±sd. from three experiments. *P<0.05, **P<0.01, ***P<0.001 and ****P<0.0001. [file 13287_2020_2093_MOESM6_ESM.jpg]

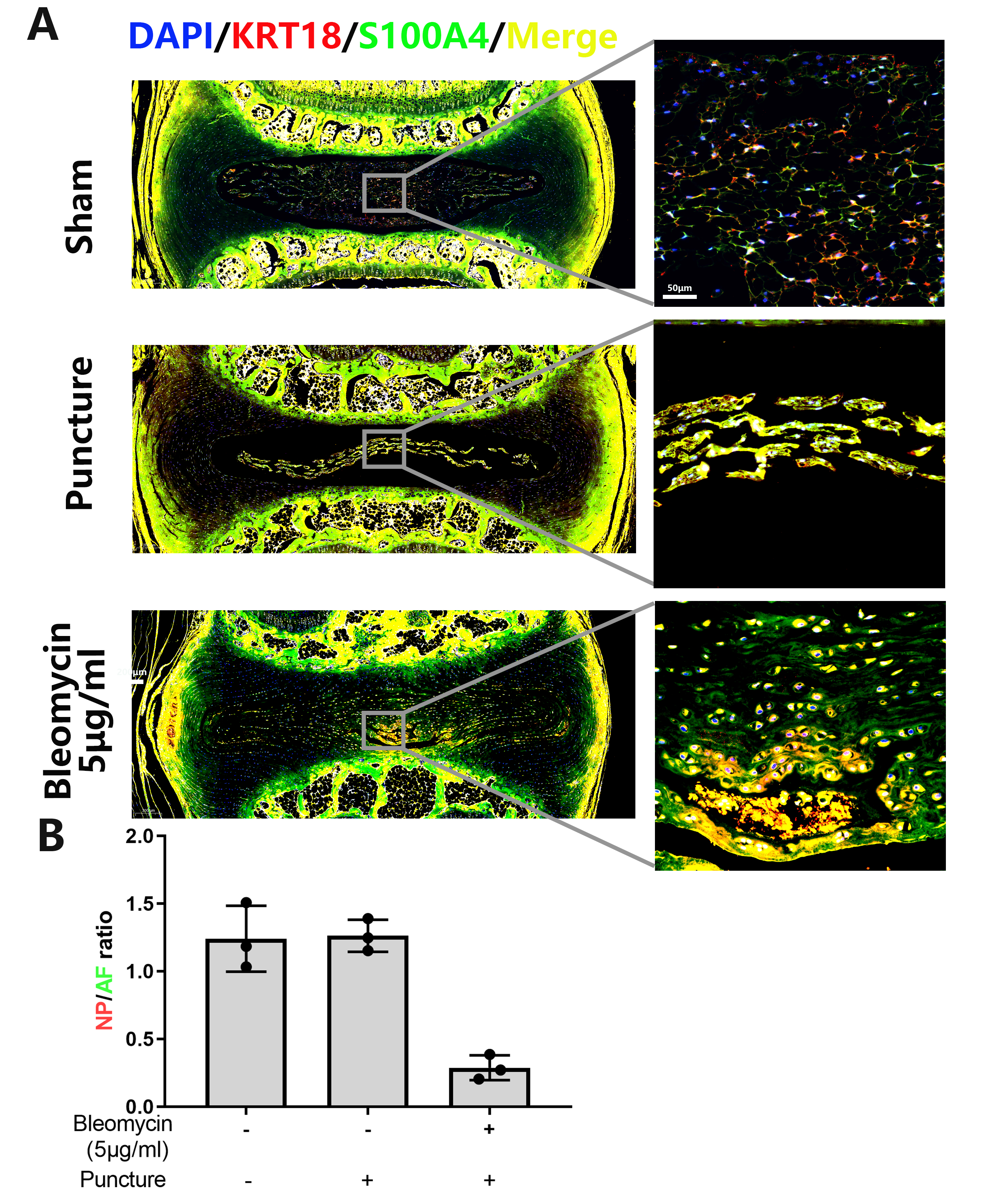

Supplement: Supplementary file 7 — Additional file 7 : Sup Figure 7. (a, c) Immunofluorescence assay of KRT18 and S100A4 in the fibrosis NP region and AF region. (b, d) The ratio IOD level between the gree region and the red region described in Sup Figure 7a were analyzed by IPP.6.0 and subsequently calculated with Graphpad8.0 by ordinary one-way ANOVA. All data are presented as mean ±sd. from three experiments. *P<0.05, **P<0.01, ***P<0.001 and ****P<0.0001. [file 13287_2020_2093_MOESM7_ESM.tif]

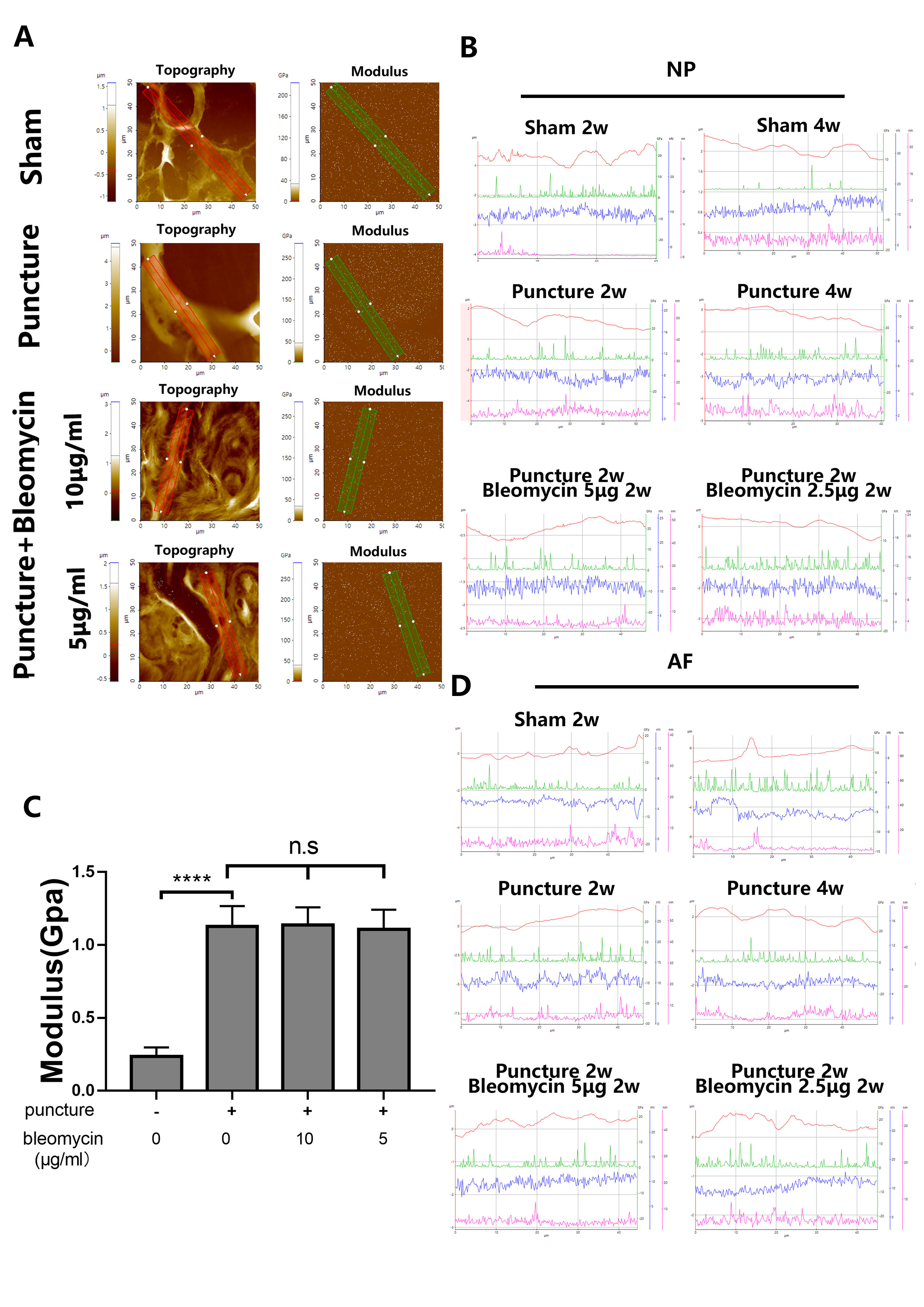

Supplement: Supplementary file 8 — Additional file 8: Sup Figure 8. (a, c) Atomic Force Microscopic of fibrosis NP region in the paraffin section mentioned in Figure 6 and Quantification of Young’s Modulus. (b, d) Evaluation of Topography-Displacement, Adhesion Force-Displacement, Young’s Modulus-Displacement and Deformation-Displacement curve. All data are presented as mean ±sd. from three experiments. *P<0.05, **P<0.01, ***P<0.001 and ****P<0.0001. [file 13287_2020_2093_MOESM8_ESM.jpg]
